# Integrated Bioinformatic Analysis of a Competing Endogenous RNA Network Reveals a Prognostic Signature in Endometrial Cancer

**DOI:** 10.3389/fonc.2019.00448

**Published:** 2019-05-29

**Authors:** Leilei Xia, Ye Wang, Qi Meng, Xiaoling Su, Jizi Shen, Jing Wang, Haiwei He, Biwei Wen, Caihong Zhang, Mingjuan Xu

**Affiliations:** ^1^Department of Obstetrics and Gynecology, Changhai Hospital, Second Military Medical University, Shanghai, China; ^2^Department of Cell Biology, Center for Stem Cell and Medicine, Second Military Medical University, Shanghai, China; ^3^Department of Urology, Changhai Hospital, Second Military Medical University, Shanghai, China; ^4^Department of Obstetrics and Gynecology, No. 455 Hospital, Second Military Medical University, Shanghai, China

**Keywords:** endometrial cancer, ceRNA network, prognostic factor, lncRNA, TCGA

## Abstract

In endometrial carcinoma, the clinical outcome directly correlates with the TNM stage, but the lack of sufficient information prevents accurate prediction. The molecular mechanism underlying the competing endogenous RNA (ceRNA) hypothesis has not been investigated in endometrial cancer. Multi-bioinformatic analyses, including differentially expressed gene analysis, ceRNA network construction, Cox regression analysis, function enrichment analysis, and protein-protein network analysis, were performed on the sequence data acquired from The Cancer Genome Atlas (TCGA) data bank. A ceRNA network comprising 366 mRNAs, 27 microRNAs (miRNAs), and 66 long non-coding RNAs (lncRNAs) was established. Survival analysis performed with the univariate Cox regression analysis revealed nine lncRNAs with prognostic power in endometrial carcinoma. In multivariate Cox regression analysis, a signature comprising LINC00491, LINC00483, ADARB2-AS1, and C8orf49 showed remarkable prognostic power. Risk score and neoplasm status, but not TNM stage, were independent prognostic factors of endometrial carcinoma. A ceRNA network comprising differentially expressed mRNAs, miRNAs, and lncRNAs may reveal the molecular events involved in the progression of endometrial carcinoma. In addition, the signature with prognostic value may discriminate patients with increased risk for poor outcome, which may allow physicians to take accurate decisions.

## Introduction

Endometrial cancer was shown to cause ~11,350 deaths in the United States this year, and its incidence has increased mainly owing to the rise in obesity, a known risk factor. Clinical outcomes directly correlate with the clinical stages, and the 5-year overall survival rate has sharply decreased from 95% in patients with stage I cancer to 16% in those with stage IV cancer (1, 2). In clinical follow-up, aside from imageological examination such as B-mode ultrasound and magnetic resonance imaging (MRI), serum tumor markers, including CA125, CA199, and CEA, may serve as the indicators for the predictive outcome in patients (3, 4). Recently, L Salmena et al. proposed that mRNAs, long non-coding RNAs (lncRNAs), and pseudogenes may regulate the expression of each other by targeting microRNAs (miRNAs) (5). lncRNAs were the biggest and most diverse class of non-coding RNAs in the human genome (6). Plenty lncRNAs play a part in pathogenesis of cancer such as unmanageable proliferation, or metastasis (7, 8), and can serve as oncogenes or antioncogenes, or by interreacting with famous oncogenes or antioncogenes such as MYC or p53, on both a transcriptional or post-transcriptional level (9, 10). Although the molecular events involved in the progression of endometrial carcinoma have been well studied, the complicated interaction between mRNAs, miRNAs, and lncRNAs that exerts crucial influence on the progression and prognosis of endometrial cancer is yet unclear.

The Cancer Genome Atlas (TCGA), a public integrated database, provides multiplatform genomic data along with the clinical information of matched patients. This database has driven the development of genomics to characterize the molecular landscape of cancers (11). Using TCGA, we analyzed differentially expressed genes, including mRNA, miRNA, and lncRNA, and constructed a lncRNA-miRNA-mRNA competing endogenous (ceRNA) network in endometrial cancer. Furthermore, we used Cox regression analysis to identify a signature based on LINC00491, LINC00483, ADARB2-AS1, and C8orf49. Of note, this signature may serve as an independent prognostic factor in endometrial cancer. This study demonstrates that these lncRNAs would allow identification of patients with endometrial cancer that are at higher risk for poor clinical outcome.

## Methods and Materials

### Data Source

All the foundation data of TCGA-UCEC project, including genetic data, transcriptome profiling, and clinical information, were acquired from the Genomic Data Commons of the National Cancer Institute (http://portal.gdc.cancer.gov). Among 587 endometrial cancer profiles, 35 were obtained from para-carcinoma tissues, while others were endometrial cancer tissues. These data were available with no restrictions for research, and this study was performed under the guidelines of TCGA. GENCODE v.27 was used to annotate RNAs in the original transcriptome profiling, and total of 19676 mRNAs, 14447 lncRNAs, 1881 miRNAs were annotated. In clinical information, overall survival data were calculated from the date of diagnosis to the date of death or last follow-up.

### Differentially Expressed mRNAs, miRNAs, and lncRNAs

The genomic data and transcriptome data from TCGA were downloaded and subjected to normalization with the calcNormFactors function with method of trimmed mean of M-values (TMM) in edgeR package. In addition, to avoid low abundance impact on the next procedure, RNAs with an average value of <1 were excluded. The differentially expressed mRNAs, miRNAs, and lncRNAs were analyzed with the exactTest function using the edgeR package. RNAs with a cutoff false discovery rate (FDR) adjusted *p* < 0.01 and |logFC| ≥ 2 were considered statistically different between cancer and normal groups (12). Heatmap was plotted using pheatmap R package.

### Construction of the ceRNA Network

According to the hypothesis of ceRNA, it is vital to match the differentially expressed mRNAs, miRNAs, and lncRNAs; thus, the network could highlight a new molecular mechanism involved in the development of endometrial cancer. Pairs of miRNA-lncRNA were establish using the miRcode database (13). Pairs of miRNA-mRNA were built using the basic data supplied by TargetScan (14), and the mRNA predicted by the database was characterized as the target mRNA and used in the subsequent step. Pearson correlation was calculated between lncRNAs and mRNAs mediated by miRNAs (15), only the pairs with coefficient >0.4 were considered may involved in ceRNA network (16). Then, to quantify the regulatory effect of lncRNA/mRNA over mRNA/lncRNA via a specific miRNA, Sumazin et al. proposed the use of conditional mutual information (17), which was calculated in JAMI software implemented in Java (18). In this analysis, pairs with a value of *p* < 0.05 was considered statistically significant.

The clusterProfiler R package created by Guang et al. (19) was used to perform functional enrichment analyses, including Gene Ontology and Kyoto Encyclopedia of Genes and Genomes (KEGG) pathway analyses. Terms with a value of *p* < 0.05 was considered statistically significant.

### Identification of a Prognostic Signature Based on the ceRNA Network

Prognostic data were created on the matrix of lncRNAs involved in the ceRNA network and matched follow-up data. Patients were classified according to the median expression of lncRNAs into high or low expression groups. Univariate Cox regression analysis was used to identify the lncRNA with prognostic value. In addition, lncRNA with a *p* < 0.05 was used in the multivariate Cox regression analysis. As the number of lncRNA was high, it is important to create a signature comprising a limited number of variables and the best Akaike information criterion (AIC). These steps in the multivariate Cox regression analysis used the function of coxph in survival R package. After the identification of the best signature that predicted the outcome of the patients with endometrial cancer, the risk score was calculated as the summation of the product of each gene and its coefficient. In addition, patients were classified into high and low risk groups with the cutoff of the median risk score. Log-rank test was used to compare the survival distribution of these two groups, as estimated by the Kaplan-Meier analysis. In addition, a receiver operating characteristic (ROC) analysis was used to estimate the predictive power of this signature using 3 years as the predicted time.

The relativity between risk score and clinical factors, including age at diagnosis, TNM stage, and neoplasm tumor status, was analyzed using the chi-square test. Both univariate and multivariate Cox regression analyses were employed to discriminate between prognostic factors in endometrial carcinoma.

### Protein-Protein Interaction Network Construction

mRNAs involved in ceRNA network based on lncRNA from the signature were subjected to protein-protein interaction network analysis using the STRING website (20).

### Statistical Analysis

Kaplan-Meier curve was conducted by SPSS 21.0 using log-rank test (21), while other statistical tests were executed by R 3.5.1 using the corresponding R package mentioned above, hazard ratios was used in Cox model (22).

## Results

### Differentially Expressed mRNAs, miRNAs, and lncRNAs

By employing differential gene expression analysis between cancer tissues and normal adjunct tissues, as per the cutoff FDR adjusted *p* < 0.01and |logFC| ≥ 2, 2,609 differentially expressed mRNAs (1,648 overexpressed and 961 down-regulated), 189 differentially expressed miRNAs (140 overexpressed and 49 down-regulated), and 1,121 differentially expressed lncRNAs (798 overexpressed and 323 down-regulated) were identified (Supplement Figure 1).

### Construction of the ceRNA Network

We constructed the ceRNA network comprising mRNAs, miRNAs, and lncRNAs. The pairs of lncRNA-miRNA were matched using miRcode, 95 lncRNAs and 27 miRNAs formed 530 potential lncRNA-miRNA pairs. The miRNA-mRNA pairs were matched based on TargetScan. As a result, 1126 pairs of lncRNAs-mRNAs have the Pearson correlation >0.4. Then, in conditional mutual information calculated in JAMI software, 1605 pairs of lncRNA-miRNA-mRNA have the *p* < 0.05 which means that in these pairs, miRNAs are mediating that interaction. Thus, the ceRNA network was completely constructed and constituted of mRNAs, miRNAs, and lncRNAs (Supplement Table 1).

It is well known that hub nodes play critical roles in biological networks. Therefore, we calculated all node degrees of the lncRNA involved in ceRNA network. According to the previously study by Han et al., in which they defined a hub as a node degree exceeding 5, we found that 15 lncRNAs could be chosen as hub nodes, and the results are shown in Supplement Figure 2.

To discover the biological terms associated with these dysregulated genes, GO and KEGG function enrichment analyses were separately performed on dysregulated mRNAs. Terms with a value of *p* < 0.05 were considered as statistically significant.

**Figure 1:**
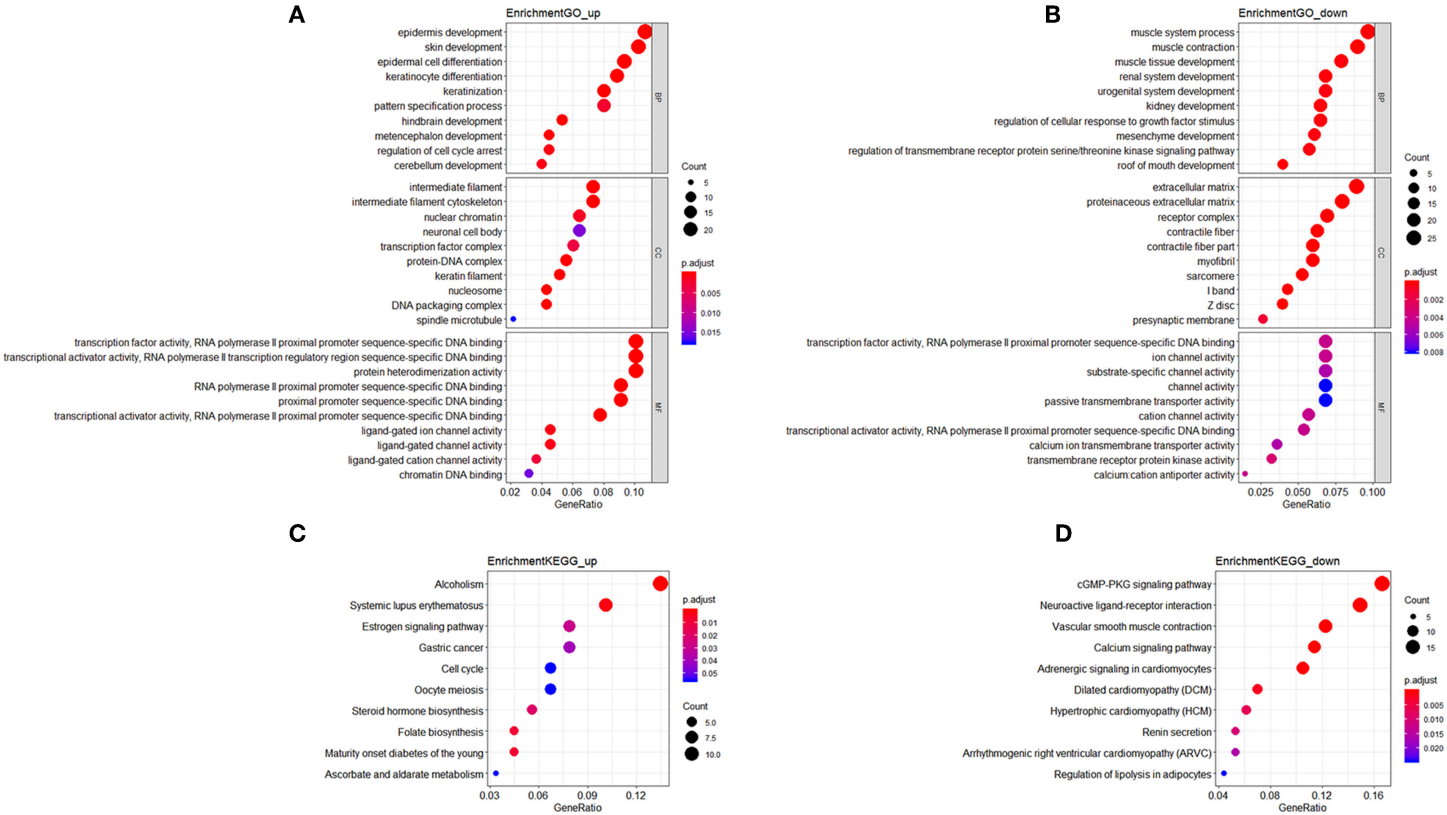
Functional enrichment analysis of mRNAs in the ceRNA network. **(A)** Gene Ontology enrichment analysis of upregulated mRNAs. **(B)** Gene Ontology enrichment analysis of downregulated mRNAs. **(C)** KEGG pathway analysis of upregulated mRNAs. **(D)** KEGG pathway analysis of downregulated mRNAs. Horizontal axis represents gene count. Vertical axis represents enrichment analysis terms. Color of each plot represents the *p* value while the size represents the gene number in this term.

In GO analysis, the overexpressed mRNAs were mainly enriched in epidermis development, intermediate filament, and transcription factor activity, RNA polymerase II proximal promoter sequence-specific DNA biding. The down-regulated mRNAs were significantly enriched in functions such as muscle system process, extracellular matrix, and transcription factor activity, RNA polymerase II proximal promoter sequence-specific DNA binding. In KEGG analysis, the overexpressed mRNAs were mainly enriched in Maturity onset diabetes of the young, and Alcoholism. The down-regulated mRNAs were mainly enriched in cGMP-PKG signaling pathway and Vascular smooth muscle contraction (Figure 1).

### Construction of Prognostic Signature Based on the ceRNA Network

**Figure 2:**
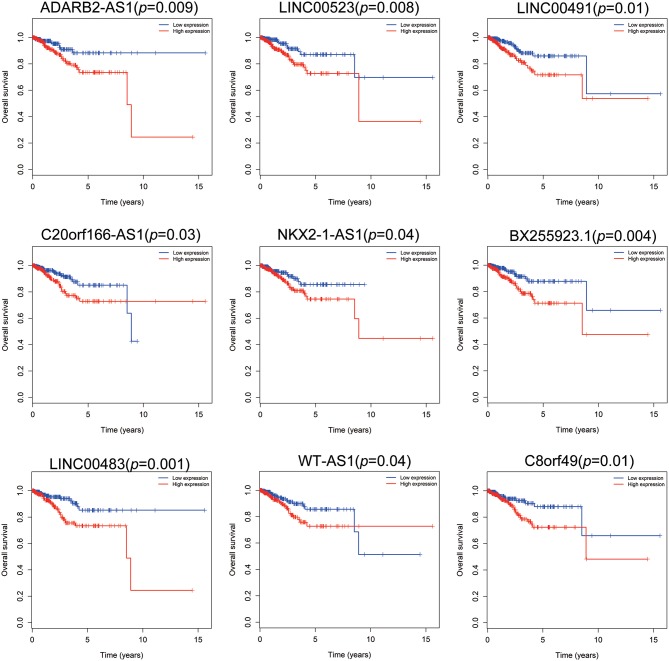
Survival analysis of nine lncRNAs. Red plot indicates overexpression, while the blue plot represents low expression. Univariate Cox regression analysis was used to identify the lncRNA with prognostic value.

**Figure 3:**
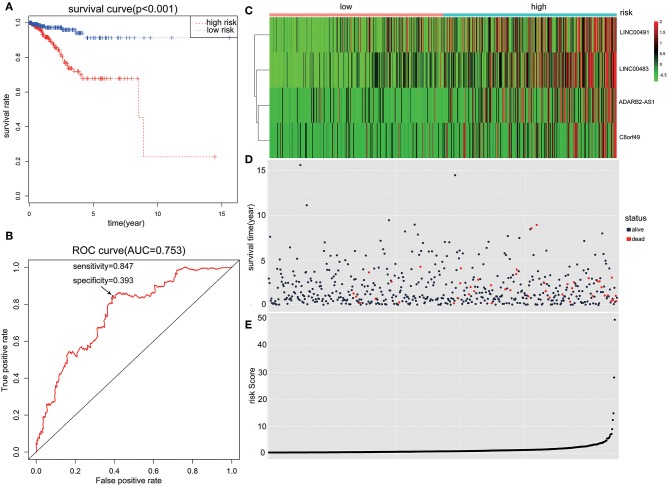
The predictive value of the risk score calculated by LINC00491, LINC00483, ADARB2-AS1, and C8orf49. **(A)** Kaplan-Meier survival analysis of the risk score for overall survival. Log-rank test was used to compare the survival distribution of these two groups. **(B)** ROC for the prediction of the 3-years survival based on risk score. Area under the curve is 0.753, the sensitivity and specificity are 0.847 and 0.393 respectively. **(C)** Heatmap of LINC00491, LINC00483, ADARB2-AS1, and C8orf49 in high-risk and low-risk groups. The color of each block represents the relative expression of lncRNA in this patient. **(D)** Survival status and survival time of each individual. Color of each plot represents the survival status of each patient. **(E)** Risk score of each individual.

Patients with incomplete clinical information (age, race, TNM stage, tumor grade, histological type, type of neoplasm, and follow-up information) were excluded from the following procedure. Univariate Cox regression analysis was applied to lncRNAs involved in the ceRNA network. Survival status and overall survival time analyses revealed nine lncRNAs with prognostic values in endometrial carcinoma (Figure 2). These nine lncRNAs were subjected to multivariate Cox regression analysis. In this step, a function of step was applied to identify the best signature that predict the outcome of patients with endometrial carcinoma. A risk score formula based on LINC00491, LINC00483, ADARB2-AS1, and C8orf49 had the lowest AIC and was selected as the best signature. The risk assessment score for the prediction of overall survival was calculated as follows: Risk score = exp_LINC00491_ × 0.13335 + exp_LINC00483_ × 0.32495 + exp_ADARB2−AS1_ × 0.25997 + exp_C8orf49_ × 0.22279. Patients were classified into two clusters using the cutoff value of the median risk score. Kaplan-Meier survival analysis indicated that the 5-year survival rates for low- and high-risk groups were more than 0.9 and 0.6, respectively (*p* < 0.001). The area under the curve in ROC analysis was 0.753, suggesting that this signature has a promising power in predicting the clinical outcome of patients with endometrial carcinoma (*p* < 0.001, sensitivity: 0.847, specificity: 0.393) (Figure 3).

Clinical information of patients with endometrial carcinoma is shown in Table 1. We used the chi-square test to estimate the correlation between risk level and other clinical factors, and found that the risk level was significantly correlated with TNM stage (*p* = 0.005), tumor grade (*p* = 0.005), histological type (*p* < 0.001), neoplasm type (*p* = 0.017), and vital status (*p* < 0.001). This finding indicates that the risk score signature was closely correlated with the above-mentioned clinical parameters (Table 2).

**Table 1:** Clinical parameters of endometrial carcinoma patients.

**Subgroup**	**Frequency**	**Percent**
Age		
< 60	160	33.9
> = 60	312	66.1
Race		
White	342	72.5
Nonwhite	130	27.5
TNM stage		
I + II	344	72.9
III–IV	128	27.1
Tumor grade		
G1 + G2	210	44.5
G3	262	55.5
Histological type		
Endometrioid endometrial adenocarcinoma	361	76.5
Other types	111	23.5
Type of neoplasm		
Tumor free	397	84.1
With tumor	75	15.9
Vital status		
Alive	432	91.5
Dead	40	8.5
Risk level		
Low	242	51.3
High	230	48.7

**Table 2:** Relationship between risk level and clinical parameters.

**Subgroup**	**Low-risk**	**High-risk**	**Total**	***P*-value**
Age				0.081
< 60	91	69	160	
> = 60	151	161	312	
Race				0.451
White	179	163	342	
Nonwhite	63	67	130	
TNM stage				0.005
I + II	190	154	344	
III–IV	52	76	128	
Tumor grade				0.005
G1 + G2	123	87	210	
G3	119	143	262	
Histological type				<0.001^*^
Endometrioid endometrial adenocarcinoma	214	147	361	
Other types	28	83	111	
Type of neoplasm				0.017
Tumor free	213	184	397	
With tumor	29	46	75	
Vital status				<0.001^*^
Alive	235	197	432	
Dead	7	33	40	

* *p < 0.05*.

**Figure 4:**
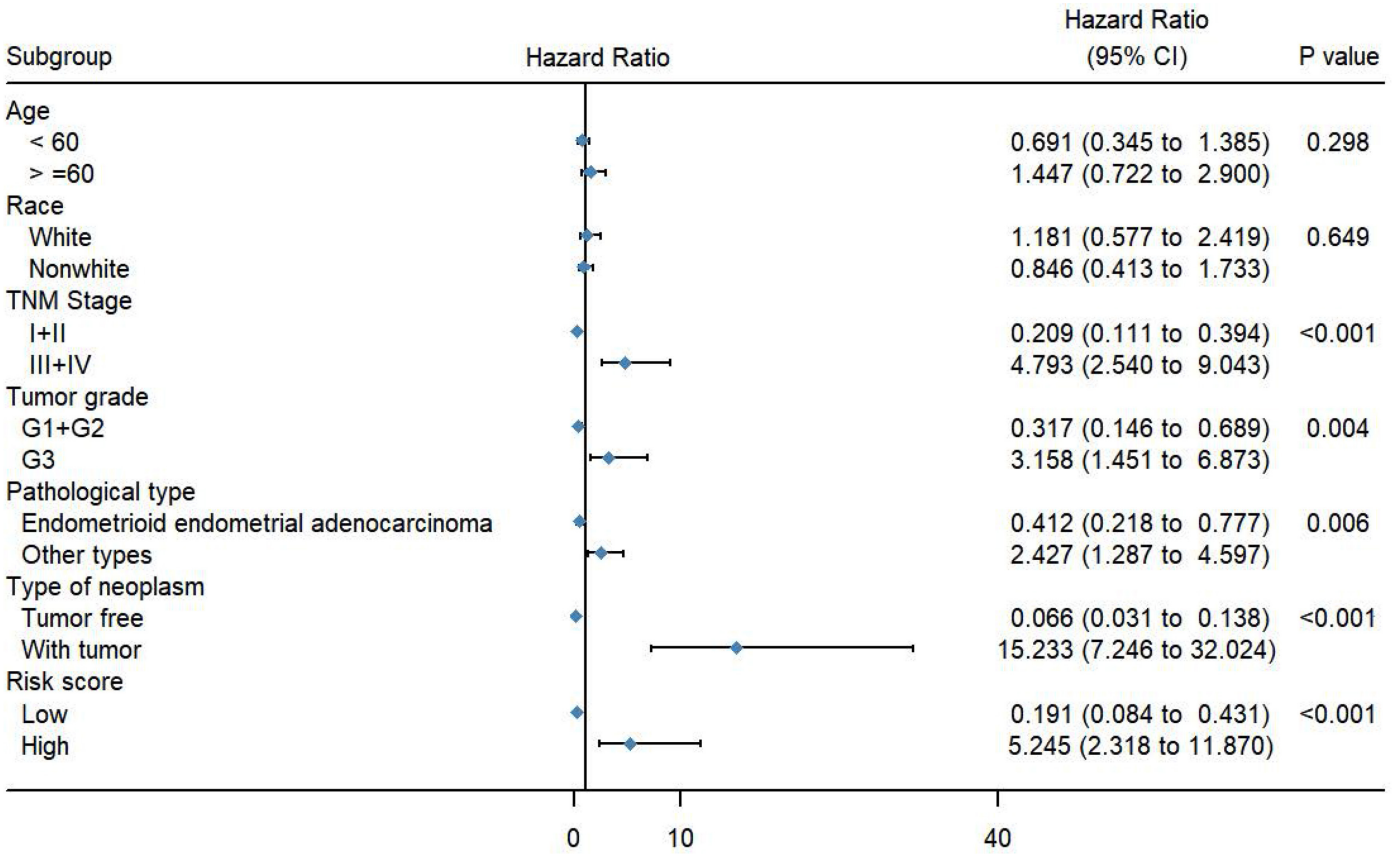
Forest map of clinical characters in univariate analysis. The coordinate of diamond represents the odds ratio. Univariate Cox regression analysis was performed. Subgroup with a value of *p* < 0.05 was considered statistically significant.

**Figure 5:**
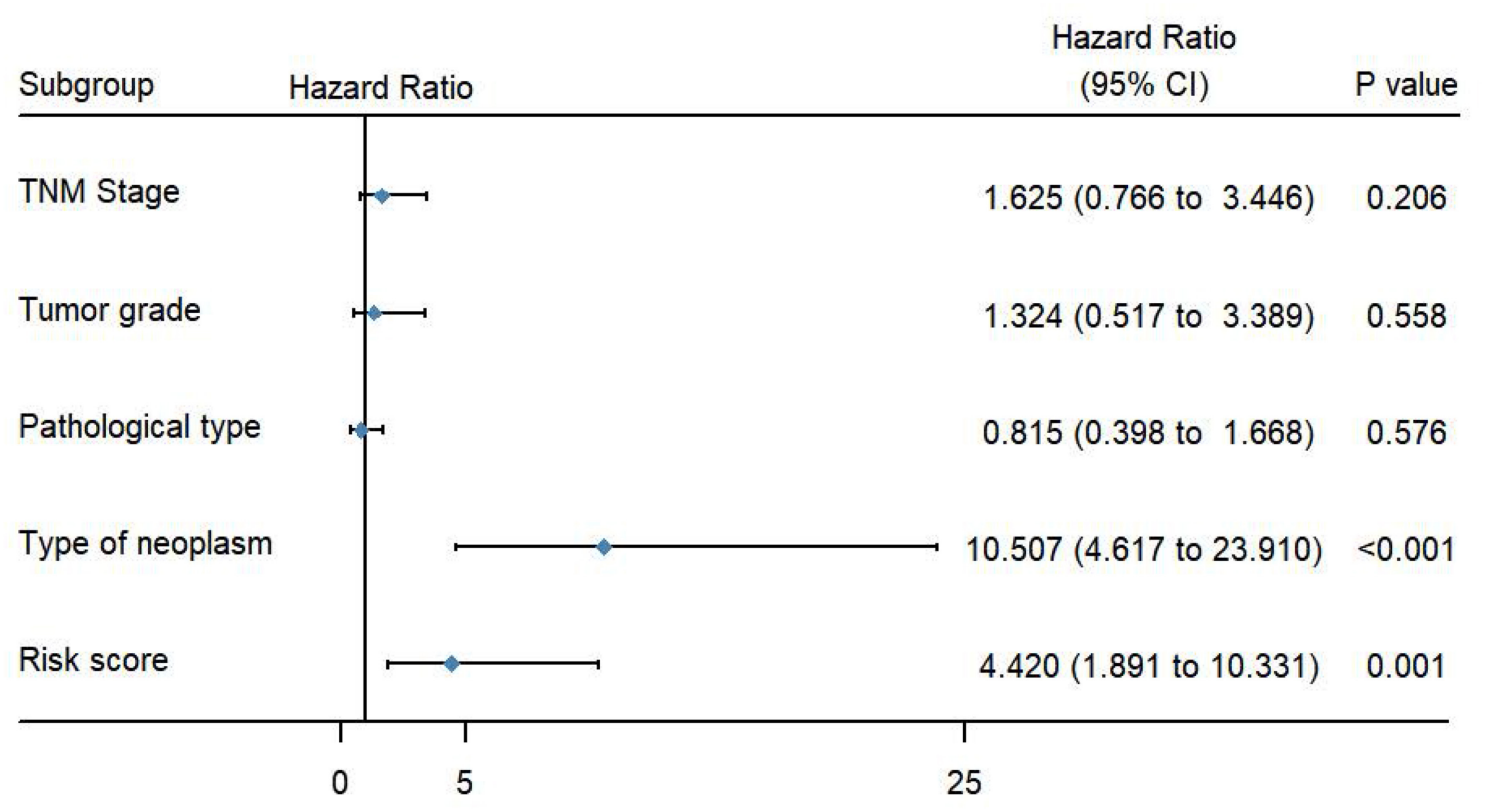
Forest map of clinical characters in multivariate analysis. The coordinate of the blue diamond represents the odds ratio. Multivariate Cox regression analysis was performed. Subgroup with a value of *p* < 0.05 was considered statistically significant.

To evaluate the predictive power of this risk signature, clinical parameters such as race, age at diagnosis, tumor grade, TNM stage, pathological type, and neoplasm type were included in the survival analysis. As shown in Figure 4, aside from tumor grade (*p* = 0.004), TNM stage (*p* < 0.001), and pathological type (*p* = 0.006), type of neoplasm (*p* < 0.001) and risk score (*p* < 0.001) were directly related to prognosis of patients. Multivariate Cox regression analysis showed that only type of neoplasm (*p* < 0.001) and risk score (*p* = 0.001), but not TNM stage (*p* = 0.206), tumor grade (*p* = 0.558), and tumor pathological type (*p* = 0.576), were statistically independent predictive factors of poorer prognosis for endometrial cancer (Figure 5).

### Protein-Protein Network Analyses

**Figure 6:**
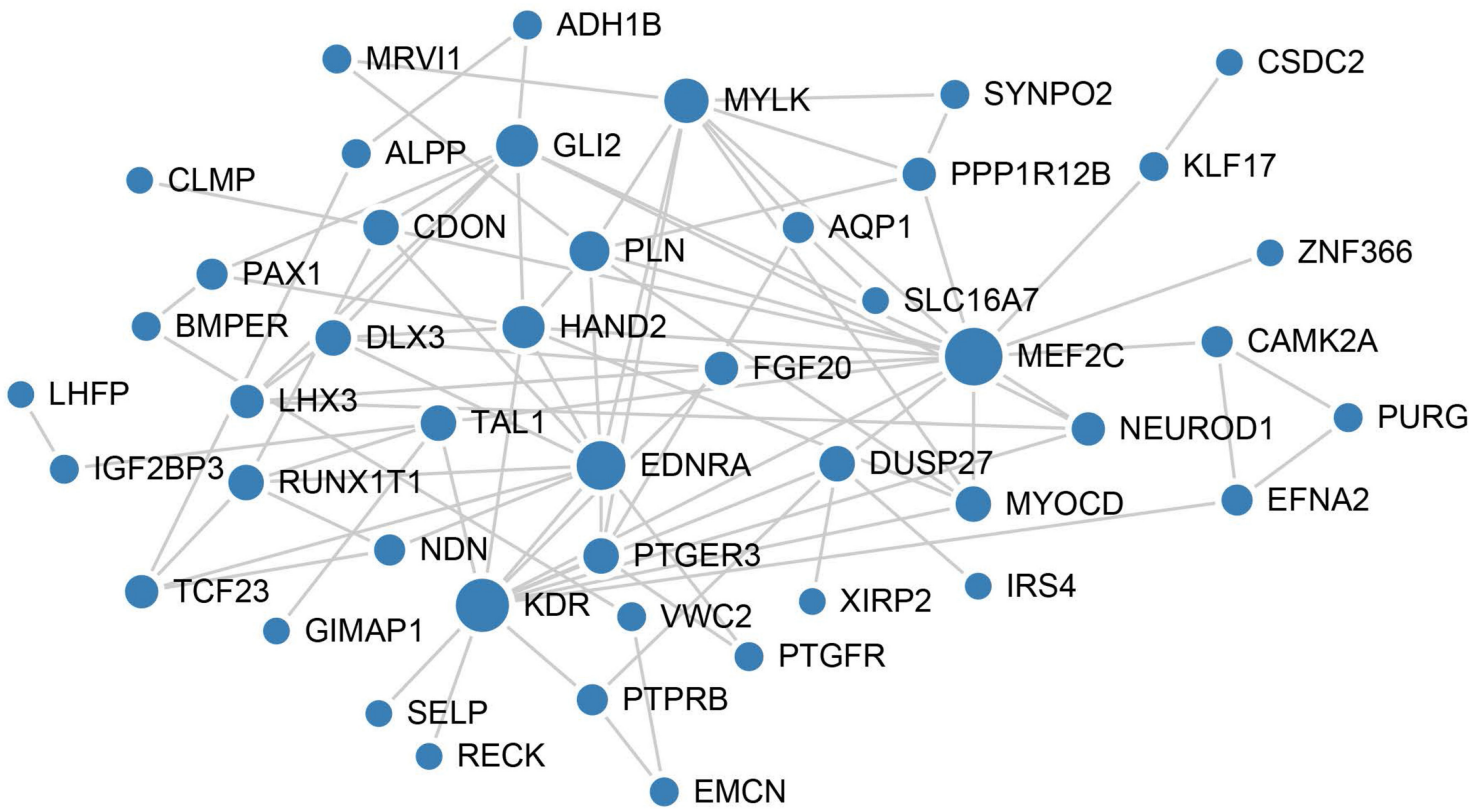
PPI network analysis of mRNAs involved in the ceRNA network of these four lncRNAs. The size of each node represents the degree of this gene.

To better understand the mechanisms underlying the function of the four lncRNAs, protein-protein interactions of mRNAs involved in the ceRNA network of these four lncRNAs were constructed using the STRING website. In this PPI network, MEF2C has the closet connection with other proteins (Figure 6).

## Discussion

Endometrial cancer is one of the three leading gynecologic tumors. The Cancer statistics of 2019 revealed 61,880 new cases and 12,160 deaths in United States. (1). Several tumor markers such as CA125, HE4, CA199, and CEA are clinically used for the diagnosis of endometrial cancer. However, the pathological process of the occurrence and development of endometrial cancer is still unclear. More precise preoperative staging and preoperative diagnosis demand better pathophysiological development and new tumor markers of endometrial cancer. Epigenetics of genes, especially lncRNAs, have been recently used for the study of endometrial cancer. Although most lncRNAs lack the capacity of coding protein, many other functions of lncRNAs have been found in endometrial cancer, including epithelial-to-mesenchymal transition (4). But due to the technical limitations, functional studies of lncRNAs are not easy in comparison with those of coding RNAs. ceRNA hypothesis provided a new solution for achieving better functional studies of lncRNAs. It proposed that lncRNA can regulate miRNA abundance by binding and sequestering them. As such, lncRNAs can regulate the expression of target mRNAs. Thus, it has been shown that an efficient way to infer the potential function of lncRNAs is by studying their relationship with miRNAs and mRNAs, whose functions have been annotated. Taken advantage of that, we mapped the ceRNA network in endometrial cancer which could provide new insights to explore the mechanism of it.

In this research, we analyzed the differentially expressed genes to develop ceRNA network and investigated the molecular events that facilitate the development of endometrial carcinoma. Using univariate and multivariate Cox regression analyses, a signature based on four lncRNAs was developed that showed promising outcomes with respect to the prediction of the patient's overall survival. This signature was closely correlated with the TNM stage and tumor grade clinical parameters and served as an independent factor, like neoplasm cancer status and unlike TNM stage. Some clinical studies have suggested the association between diabetes as well as hypertension with the outcome of patients with endometrial cancer (23, 24); however, we did not perform statistical analysis of diabetes and hypertension in the present study, as more than 50% values were missing.

In general, the survival outcome for patients with endometrial cancer is mainly predicted by two elements, namely, TNM stage and type of neoplasm, but a quantifiable index is lacking.

Several studies have shown that the expression of hormone receptors such as estrogen and progesterone receptors is a favorable independent prognostic factor (25, 26). In addition, hormonal therapy was considered as a supplemental therapy in clinical setting. Mutation of the well-known gene P53 is associated with poor clinical outcome, and the overall survival of patients with endometrial cancer with alterations in p53 gene expression was much lower than that of patients with the wild-type p53 (27). Moreover, the overexpression of human epidermal growth factor receptor 2 (HER2) gene is an independent prognostic factor associated with poor overall survival (28).

An efficient and sensitive marker that predicts the outcome of endometrial cancer is still lacking. In this study, we identified a signature based on LINC00491, LINC00483, ADARB2-AS1, and C8orf49 to discriminate patients with endometrial cancer that are at increased risk for poor outcome in combination with the information related to TNM stage and type of neoplasm.

The results of this analysis showed that the expressions of four lncRNAs (LINC00491, LINC00483, ADARB2-AS1, and C8orf49) were markedly different between endometrial cancer tissues and normal endometrial tissues. Many of these genes are incompletely studied. C8orf49 is also called as GATA4 downstream membrane gene (G4DM). GATA4 (GATA transcription factor 4) is a zinc-finger transcription factor involved in the development of heart and adult cardiomyocytes. Mutations in GATA4 gene may cause congenital heart diseases (29) such as the tetralogy of Fallot, atrial septal defect, ventricular septal defect, atrioventricular septal defect, and dilated cardiomyopathy. A key feature of GATA4 is its two-zinc finger domain, which binds to the specific region of the target gene. In serval cancer types, GATA4 serves as a potential tumor suppressor, and hypermethylation and hypomethylation of GATA4 are closely related to the malignant behavior of cancers (30, 31). GATA4 may also be used as a biomarker for ovarian cancer (32). The expression of GATA4 may change during cardiocyte differentiation through the effect of the transcription of the target gene. GATA4 gene is also expressed in the uterus, and C8orf49 is one of the target genes of GATA4. Therefore, C8orf49 may play an important role in the differentiation of endometrial cancer cells. In addition, studies have indicated that ADARB2-AS1, as an open-reading frame, may contribute to the risk of pancreatic ductal adenocarcinoma (PDAC). Along with other seven lncRNAs, ADARB2-AS1 showed better accuracy than the standard clinical and radiologic features in distinguishing aggressive/malignant IPMNs (33). However, the underlying mechanism is unclear.

It is well known that hub genes play critical roles in biological networks. Therefore, node degrees of lncRNA involved in this ceRNA network were calculated. A lncRNA with a node degree >5 was considered as hub lncRNA. In this study, total of 15 lncRNAs were identified with high degree in the ceRNA network. C8orf49 which has prognostic value also act as hub lncRNA in endometrial cancer. This suggests that C8orf49 may play critical roles in the origin and development of endometrial cancer. Here, we demonstrate for the first time the construction of a ceRNA network in endometrial cancer to reveal the molecular mechanism that facilitates the development of endometrial cancer. A signature based on LINC00491, LINC00483, ADARB2-AS1, and C8orf49 was identified as a biomarker to discriminate between patients with high and poor risk outcome. The lncRNAs involved in this signature may serve as therapeutic targets for precision medicine in endometrial cancer. Further studies are warranted to explore the biological function and reveal the molecular mechanism underlying the role of LINC00491, LINC00483, ADARB2-AS1, and C8orf49 in endometrial cancer.

## Conclusion

This study focused on a ceRNA network to provide a novel perspective and insight into endometrial cancer and suggested that the signature based on LINC00491, LINC00483, ADARB2-AS1, and C8orf49 could serve as an independent prognostic biomarker in endometrial cancer.

## Ethics Statement

High-throughput sequencing-counts (HTSeq-counts) and miRNA sequencing profiles were obtained from the TCGA data portal, which is a publicly available dataset. Therefore, no ethics approval is needed.

## Author Contributions

LX constructed ceRNA network using R software. QM analyzed the data using SPSS. YW download data from TCGA. XS and BW used photoshop and illustration software. JS and CZ organized data. JW perform GO and KEGG analysis. HH perform PPI network. MX designed this study.

### Conflict of Interest Statement

The authors declare that the research was conducted in the absence of any commercial or financial relationships that could be construed as a potential conflict of interest.

## Supplementary Material

Supplement Figure 1 Heatmap of differentially expressed RNAs. The one on the left is the heatmap of 50 randomly selected differentially expressed mRNAs. The center one is the heatmap of 50 differentially expressed lncRNAs, while the one on the right is the heatmap of 50 randomly selected differentially expressed miRNAs. Orange indicates high-level RNA expression, whereas blue indicates low expression. The first row of each map is the type of each sample. Pink represents normal samples, while blue represents cancerous samples.

Supplement Figure 2 All lncRNA node degree analysis reveals specific properties of the ceRNA network.

Supplement Table 1 ceRNA network of endometrial cancer.
