# Supplementary material for: Integrated Bioinformatic Analysis of a Competing Endogenous RNA Network Reveals a Prognostic Signature in Endometrial Cancer
Source: Front Oncol. 2019 May 29;9:448. doi: 10.3389/fonc.2019.00448 (PMC6549402; doi:10.3389/fonc.2019.00448)
Supplement: Supplement Figure 1 — Heatmap of differentially expressed RNAs. The one on the left is the heatmap of 50 randomly selected differentially expressed mRNAs. The center one is the heatmap of 50 differentially expressed lncRNAs, while the one on the right is the heatmap of 50 randomly selected differentially expressed miRNAs. Orange indicates high-level RNA expression, whereas blue indicates low expression. The first row of each map is the type of each sample. Pink represents normal samples, while blue represents cancerous samples. [file Table_1.docx]

Supplement Table 1: ceRNA network of endometrial cancer

| gene1 | gene2 | miRNA | CMI | p-value |
| --- | --- | --- | --- | --- |
| ANGPTL1 | DLX6-AS1 | hsa-mir-141 | 0.022099 | 0.049 |
| BRS3 | LINC00237 | hsa-mir-205 | 0.014381 | 0.049 |
| CHRDL1 | LINC00473 | hsa-mir-195 | 0.021636 | 0.049 |
| CLRN1-AS1 | TSHZ3 | hsa-mir-489 | 0.014588 | 0.049 |
| CMAHP | AKAP2 | hsa-mir-424 | 0.020212 | 0.049 |
| GPRASP1 | C15orf54 | hsa-mir-301b | 0.013435 | 0.049 |
| KIAA0087 | RECK | hsa-mir-141 | 0.070008 | 0.049 |
| KLF17 | MAGI2-AS3 | hsa-mir-141 | 0.066887 | 0.049 |
| LINC00355 | JPH4 | hsa-mir-195 | 0.04908 | 0.049 |
| MEG3 | ADRB3 | hsa-mir-211 | 0.017877 | 0.049 |
| MIR210HG | RTKN2 | hsa-mir-122 | 0.02398 | 0.049 |
| MIR210HG | WNT2B | hsa-mir-195 | 0.029001 | 0.049 |
| NAV2-AS2 | MYLK | hsa-mir-182 | 0.032421 | 0.049 |
| OLFML2A | LINC00314 | hsa-mir-143 | 0.023732 | 0.049 |
| PRKG1 | LINC00337 | hsa-mir-383 | 0.013098 | 0.049 |
| RAB23 | LINC00470 | hsa-mir-143 | 0.020953 | 0.049 |
| TGFB1I1 | LINC00314 | hsa-mir-143 | 0.025139 | 0.049 |
| TPH1 | MEG3 | hsa-mir-215 | 0.020488 | 0.049 |
| WT1-AS | PCSK2 | hsa-mir-424 | 0.018309 | 0.049 |
| WT1-AS | TCF23 | hsa-mir-106a | 0.025021 | 0.049 |
| WT1-AS | AKAP2 | hsa-mir-145 | 0.028668 | 0.049 |
| AKT3 | LINC00211 | hsa-mir-204 | 0.015171 | 0.048 |
| C20orf166-AS1 | GUCY1A2 | hsa-mir-183 | 0.031373 | 0.048 |
| DPYSL5 | KIAA0087 | hsa-mir-183 | 0.0271 | 0.048 |
| HRH3 | LINC00458 | hsa-mir-205 | 0.022876 | 0.048 |
| LINC00355 | TCF23 | hsa-mir-195 | 0.037256 | 0.048 |
| LINC00470 | GRM7 | hsa-mir-424 | 0.063729 | 0.048 |
| LINC00491 | TAL1 | hsa-mir-145 | 0.147079 | 0.048 |
| MAMDC2 | MEG3 | hsa-mir-508 | 0.012274 | 0.048 |
| MEG3 | LSAMP | hsa-mir-215 | 0.019708 | 0.048 |
| MEG3 | MAMDC2 | hsa-mir-508 | 0.016983 | 0.048 |
| MEG3 | CLSTN2 | hsa-mir-215 | 0.029223 | 0.048 |
| MYOCD | LINC00488 | hsa-mir-205 | 0.012303 | 0.048 |
| NKX2-1-AS1 | SCRG1 | hsa-mir-145 | 0.079069 | 0.048 |
| PLN | LINC00393 | hsa-mir-106a | 0.020372 | 0.048 |
| TMEM196 | WT1-AS | hsa-mir-145 | 0.125649 | 0.048 |
| AL713998.1 | RBP2 | hsa-mir-383 | 0.017143 | 0.047 |
| ASPA | ADAMTS9-AS2 | hsa-mir-205 | 0.023126 | 0.047 |
| B3GNT4 | AGAP11 | hsa-mir-211 | 0.036896 | 0.047 |
| DDR2 | LINC00052 | hsa-mir-489 | 0.023487 | 0.047 |
| DIO3OS | PRLHR | hsa-mir-143 | 0.046571 | 0.047 |
| DPYSL5 | RMST | hsa-mir-211 | 0.028829 | 0.047 |
| GPR12 | DIO3OS | hsa-mir-211 | 0.013835 | 0.047 |
| KLHL4 | KIAA0087 | hsa-mir-141 | 0.016139 | 0.047 |
| KRT85 | JAZF1-AS1 | hsa-mir-216b | 0.02059 | 0.047 |
| LINC00470 | JPH4 | hsa-mir-143 | 0.143774 | 0.047 |
| LINC00483 | LHFP | hsa-mir-215 | 0.021137 | 0.047 |
| LINC00488 | SORBS1 | hsa-mir-205 | 0.024871 | 0.047 |
| LSAMP | LINC00488 | hsa-mir-96 | 0.015336 | 0.047 |
| MIR205HG | KRT12 | hsa-mir-143 | 0.021317 | 0.047 |
| MSRB3 | LINC00355 | hsa-mir-195 | 0.024212 | 0.047 |
| NEUROD1 | LINC00483 | hsa-mir-216b | 0.029091 | 0.047 |
| TMEM196 | DIO3OS | hsa-mir-143 | 0.038528 | 0.047 |
| WT1-AS | TNS1 | hsa-mir-363 | 0.024282 | 0.047 |
| BRS3 | C10orf126 | hsa-mir-363 | 0.024266 | 0.046 |
| C10orf126 | MTUS2 | hsa-mir-140 | 0.014744 | 0.046 |
| C2orf48 | MAGI2 | hsa-mir-424 | 0.049703 | 0.046 |
| CAMK2A | LINC00314 | hsa-mir-143 | 0.025024 | 0.046 |
| CMAHP | UGT1A10 | hsa-mir-205 | 0.018268 | 0.046 |
| HCG23 | PODN | hsa-mir-106a | 0.018626 | 0.046 |
| HRH3 | CMAHP | hsa-mir-183 | 0.01613 | 0.046 |
| JAZF1-AS1 | KRT85 | hsa-mir-216b | 0.032931 | 0.046 |
| JAZF1-AS1 | BPIFA1 | hsa-mir-143 | 0.015721 | 0.046 |
| KRTAP3-3 | DNM3OS | hsa-mir-145 | 0.106486 | 0.046 |
| LINC00488 | MAMDC2 | hsa-mir-205 | 0.020845 | 0.046 |
| LRRN4CL | MIR205HG | hsa-mir-215 | 0.027968 | 0.046 |
| MAGI2-AS3 | KLF17 | hsa-mir-425 | 0.041962 | 0.046 |
| MYOCD | WT1-AS | hsa-mir-363 | 0.022179 | 0.046 |
| PTGFR | LINC00470 | hsa-mir-143 | 0.02138 | 0.046 |
| SORBS1 | LINC00488 | hsa-mir-205 | 0.013431 | 0.046 |
| SVIL | LINC00314 | hsa-mir-143 | 0.025474 | 0.046 |
| ZEB1 | AL713998.1 | hsa-mir-141 | 0.018145 | 0.046 |
| ADAMTS9-AS2 | PGM5 | hsa-mir-205 | 0.012124 | 0.045 |
| AGAP11 | KCTD12 | hsa-mir-200a | 0.025517 | 0.045 |
| AGAP11 | DPYSL5 | hsa-mir-216b | 0.01668 | 0.045 |
| AKT3 | MAGI2-AS3 | hsa-mir-210 | 0.054546 | 0.045 |
| BRS3 | MUC2 | hsa-mir-383 | 0.015612 | 0.045 |
| C20orf166-AS1 | CXorf36 | hsa-mir-183 | 0.029357 | 0.045 |
| CMAHP | KIAA1462 | hsa-mir-424 | 0.055394 | 0.045 |
| CNTN4 | NOVA1-AS1 | hsa-mir-425 | 0.019693 | 0.045 |
| CXorf36 | C20orf166-AS1 | hsa-mir-489 | 0.019007 | 0.045 |
| DDR2 | NAV2-AS2 | hsa-mir-182 | 0.021348 | 0.045 |
| DSCR10 | HNF4A | hsa-mir-424 | 0.03186 | 0.045 |
| FAM9B | C15orf54 | hsa-mir-182 | 0.012733 | 0.045 |
| GLP2R | LINC00314 | hsa-mir-106a | 0.014675 | 0.045 |
| LINC00211 | HIST1H2AG | hsa-mir-183 | 0.013811 | 0.045 |
| LINC00458 | JAZF1 | hsa-mir-122 | 0.024359 | 0.045 |
| LINC00458 | KCNJ13 | hsa-mir-143 | 0.019701 | 0.045 |
| LINC00470 | KLHL1 | hsa-mir-195 | 0.053544 | 0.045 |
| LINC00483 | EFNA2 | hsa-mir-216b | 0.02123 | 0.045 |
| LMOD1 | LINC00458 | hsa-mir-122 | 0.02879 | 0.045 |
| MIR205HG | SLC35F1 | hsa-mir-122 | 0.028227 | 0.045 |
| MYCT1 | LINC00237 | hsa-mir-143 | 0.018069 | 0.045 |
| MYCT1 | MAGI2-AS3 | hsa-mir-210 | 0.059748 | 0.045 |
| PODN | HCG23 | hsa-mir-106a | 0.030223 | 0.045 |
| PTGFR | WT1-AS | hsa-mir-145 | 0.128386 | 0.045 |
| ROBO4 | C20orf166-AS1 | hsa-mir-489 | 0.018006 | 0.045 |
| STXBP5L | NKX2-1-AS1 | hsa-mir-145 | 0.034323 | 0.045 |
| SYNPO2 | LINC00355 | hsa-mir-195 | 0.026998 | 0.045 |
| AGAP11 | HOXC13 | hsa-mir-195 | 0.035819 | 0.044 |
| AXL | C20orf166-AS1 | hsa-mir-489 | 0.020135 | 0.044 |
| C8orf49 | SEZ6L | hsa-mir-100 | 0.034135 | 0.044 |
| CMAHP | STXBP5L | hsa-mir-205 | 0.015619 | 0.044 |
| DLX6-AS1 | LMOD1 | hsa-mir-145 | 0.380403 | 0.044 |
| GPR22 | WT1-AS | hsa-mir-145 | 0.124249 | 0.044 |
| LINC00314 | JPH2 | hsa-mir-143 | 0.17353 | 0.044 |
| LINC00393 | RERG | hsa-mir-106a | 0.016423 | 0.044 |
| LINC00470 | PBX1 | hsa-mir-429 | 0.020736 | 0.044 |
| PRKG1 | WT1-AS | hsa-mir-145 | 0.124506 | 0.044 |
| SORCS3 | C10orf126 | hsa-mir-200a | 0.024387 | 0.044 |
| C20orf166-AS1 | PLN | hsa-mir-183 | 0.032352 | 0.043 |
| CLDN19 | RMST | hsa-mir-204 | 0.030574 | 0.043 |
| CXorf36 | LINC00491 | hsa-mir-145 | 0.017878 | 0.043 |
| DLX3 | LINC00483 | hsa-mir-96 | 0.01899 | 0.043 |
| DLX6-AS1 | TAGLN3 | hsa-mir-195 | 0.016915 | 0.043 |
| DPYSL5 | RMST | hsa-mir-204 | 0.032071 | 0.043 |
| GPR88 | RMST | hsa-mir-211 | 0.030298 | 0.043 |
| HOXC12 | AGAP11 | hsa-mir-195 | 0.01695 | 0.043 |
| LIFR | C2orf48 | hsa-mir-106a | 0.01326 | 0.043 |
| MEG3 | KIAA1462 | hsa-mir-205 | 0.018697 | 0.043 |
| MYCT1 | BX255923.1 | hsa-mir-211 | 0.04461 | 0.043 |
| PLN | C20orf166-AS1 | hsa-mir-183 | 0.027732 | 0.043 |
| PPP1R12B | AL713998.1 | hsa-mir-141 | 0.018608 | 0.043 |
| SYDE1 | LINC00237 | hsa-mir-143 | 0.016461 | 0.043 |
| ABCG2 | BX255923.1 | hsa-mir-211 | 0.045557 | 0.042 |
| C1QTNF7 | LINC00470 | hsa-mir-424 | 0.019729 | 0.042 |
| CBX7 | DIO3OS | hsa-mir-143 | 0.039126 | 0.042 |
| DIO3OS | CNRIP1 | hsa-mir-143 | 0.083196 | 0.042 |
| GPR22 | AL713998.1 | hsa-mir-141 | 0.019481 | 0.042 |
| IFFO1 | LINC00237 | hsa-mir-143 | 0.017622 | 0.042 |
| LINC00458 | L1CAM | hsa-mir-141 | 0.040771 | 0.042 |
| LINC00488 | DGKB | hsa-mir-122 | 0.017439 | 0.042 |
| NOVA1-AS1 | NECAB1 | hsa-mir-425 | 0.028765 | 0.042 |
| PFKFB4 | CMAHP | hsa-mir-145 | 0.079946 | 0.042 |
| TMEM200B | LINC00211 | hsa-mir-204 | 0.013554 | 0.042 |
| VWC2 | C2orf48 | hsa-mir-424 | 0.017181 | 0.042 |
| C20orf166-AS1 | CBX7 | hsa-mir-183 | 0.0398 | 0.041 |
| CDON | LINC00458 | hsa-mir-200a | 0.017469 | 0.041 |
| CMAHP | KCTD4 | hsa-mir-200a | 0.020516 | 0.041 |
| CMAHP | HSD17B13 | hsa-mir-205 | 0.01925 | 0.041 |
| DIO3OS | CSDC2 | hsa-mir-143 | 0.10862 | 0.041 |
| FOXL2 | LINC00237 | hsa-mir-143 | 0.017024 | 0.041 |
| LINC00337 | TSHZ3 | hsa-mir-383 | 0.040094 | 0.041 |
| MEG3 | JAZF1 | hsa-mir-301b | 0.056964 | 0.041 |
| PLN | MEG3 | hsa-mir-205 | 0.024831 | 0.041 |
| WT1 | MAGI2-AS3 | hsa-mir-429 | 0.063422 | 0.041 |
| WT1-AS | TSPAN2 | hsa-mir-363 | 0.023577 | 0.041 |
| AKT3 | LINC00314 | hsa-mir-143 | 0.025971 | 0.04 |
| AL713998.1 | PPP1R12B | hsa-mir-141 | 0.037309 | 0.04 |
| ATXN8OS | DPYSL5 | hsa-mir-211 | 0.015152 | 0.04 |
| C20orf166-AS1 | LMOD1 | hsa-mir-183 | 0.026922 | 0.04 |
| DNM3OS | KRTAP3-3 | hsa-mir-145 | 0.034489 | 0.04 |
| DPP6 | MAGI2-AS3 | hsa-mir-106a | 0.016186 | 0.04 |
| GUCY1A2 | BX255923.1 | hsa-mir-211 | 0.045095 | 0.04 |
| KRT12 | MIR205HG | hsa-mir-143 | 0.020367 | 0.04 |
| LINC00314 | TMEM196 | hsa-mir-143 | 0.023832 | 0.04 |
| LINC00483 | SYNPO2 | hsa-mir-215 | 0.0158 | 0.04 |
| LMOD1 | C20orf166-AS1 | hsa-mir-489 | 0.020797 | 0.04 |
| MAMDC2 | DLX6-AS1 | hsa-mir-211 | 0.012127 | 0.04 |
| MEF2C | C2orf48 | hsa-mir-106a | 0.014956 | 0.04 |
| MYH11 | AGAP11 | hsa-mir-424 | 0.035026 | 0.04 |
| PCSK2 | POU6F2-AS2 | hsa-mir-383 | 0.032063 | 0.04 |
| PPP1R12B | NAV2-AS2 | hsa-mir-182 | 0.022907 | 0.04 |
| PSD | MEG3 | hsa-mir-301b | 0.021967 | 0.04 |
| SVEP1 | JAZF1-AS1 | hsa-mir-205 | 0.024546 | 0.04 |
| TCEAL7 | LINC00237 | hsa-mir-143 | 0.018176 | 0.04 |
| TGFB1I1 | ADAMTS9-AS2 | hsa-mir-96 | 0.024692 | 0.04 |
| WT1 | MIR205HG | hsa-mir-143 | 0.018047 | 0.04 |
| ZCCHC24 | NOVA1-AS1 | hsa-mir-425 | 0.020477 | 0.04 |
| BX255923.1 | IRS4 | hsa-mir-211 | 0.022859 | 0.039 |
| C2orf48 | OSR2 | hsa-mir-424 | 0.093277 | 0.039 |
| CMAHP | TCF23 | hsa-mir-424 | 0.034663 | 0.039 |
| DIO3OS | AGTR1 | hsa-mir-211 | 0.022926 | 0.039 |
| DLX6-AS1 | TMEM200A | hsa-mir-211 | 0.018504 | 0.039 |
| GPIHBP1 | LINC00473 | hsa-mir-195 | 0.022956 | 0.039 |
| KDR | C20orf166-AS1 | hsa-mir-183 | 0.027645 | 0.039 |
| LINC00494 | AKAP2 | hsa-mir-508 | 0.020923 | 0.039 |
| LSAMP-AS1 | HOXD12 | hsa-mir-183 | 0.041505 | 0.039 |
| MAGI2-AS3 | RERG | hsa-mir-210 | 0.050623 | 0.039 |
| MRGPRF | LINC00458 | hsa-mir-122 | 0.028872 | 0.039 |
| MYCT1 | C8orf49 | hsa-mir-143 | 0.026476 | 0.039 |
| NAALAD2 | LINC00052 | hsa-mir-489 | 0.02334 | 0.039 |
| SNAP25 | LINC00355 | hsa-mir-141 | 0.019717 | 0.039 |
| TCEAL7 | LINC00211 | hsa-mir-204 | 0.014818 | 0.039 |
| VGLL3 | MEG3 | hsa-mir-215 | 0.023604 | 0.039 |
| ADAMTS9-AS2 | OSR2 | hsa-mir-96 | 0.024156 | 0.038 |
| CLDN19 | LINC00443 | hsa-mir-363 | 0.019439 | 0.038 |
| EFNA2 | KIAA0087 | hsa-mir-183 | 0.0274 | 0.038 |
| KLF2 | LINC00470 | hsa-mir-424 | 0.020473 | 0.038 |
| KRTAP4-4 | C10orf126 | hsa-mir-363 | 0.023788 | 0.038 |
| LHX3 | FRMD6-AS2 | hsa-mir-184 | 0.0191 | 0.038 |
| LINC00355 | CHRNA4 | hsa-mir-122 | 0.024066 | 0.038 |
| MIR205HG | LRRN4CL | hsa-mir-215 | 0.020188 | 0.038 |
| MRGPRF | WT1-AS | hsa-mir-363 | 0.020857 | 0.038 |
| MYOCD | WT1-AS | hsa-mir-145 | 0.125252 | 0.038 |
| NAV2-AS2 | CNTN4 | hsa-mir-182 | 0.017493 | 0.038 |
| PBX1 | LINC00523 | hsa-mir-508 | 0.016295 | 0.038 |
| RSPO3 | CMAHP | hsa-mir-215 | 0.019369 | 0.038 |
| SLC16A7 | NAV2-AS2 | hsa-mir-182 | 0.022433 | 0.038 |
| TCF23 | BX255923.1 | hsa-mir-211 | 0.047466 | 0.038 |
| TMEM132D | C10orf126 | hsa-mir-140 | 0.01946 | 0.038 |
| TNFAIP8L3 | LINC00488 | hsa-mir-205 | 0.013187 | 0.038 |
| VSTM4 | LINC00470 | hsa-mir-424 | 0.018672 | 0.038 |
| AGAP11 | EMILIN1 | hsa-mir-200a | 0.112971 | 0.037 |
| CMAHP | CBX7 | hsa-mir-424 | 0.037229 | 0.037 |
| CXCL12 | C2orf48 | hsa-mir-424 | 0.015704 | 0.037 |
| DES | LINC00355 | hsa-mir-195 | 0.026179 | 0.037 |
| DPP6 | LINC00470 | hsa-mir-424 | 0.021724 | 0.037 |
| GPRASP2 | RMST | hsa-mir-195 | 0.021038 | 0.037 |
| GUCY1A2 | C2orf48 | hsa-mir-424 | 0.016591 | 0.037 |
| GUCY1A2 | LINC00237 | hsa-mir-143 | 0.017277 | 0.037 |
| IRS4 | DIO3OS | hsa-mir-143 | 0.040337 | 0.037 |
| LINC00314 | PLN | hsa-mir-143 | 0.268107 | 0.037 |
| LINC00355 | GAD2 | hsa-mir-424 | 0.043679 | 0.037 |
| LINC00488 | TNFAIP8L3 | hsa-mir-205 | 0.032657 | 0.037 |
| MYLK | RMST | hsa-mir-195 | 0.018606 | 0.037 |
| PLSCR4 | NAV2-AS2 | hsa-mir-182 | 0.023447 | 0.037 |
| SYT1 | MEG3 | hsa-mir-215 | 0.026151 | 0.037 |
| T | NKX2-1-AS1 | hsa-mir-145 | 0.035124 | 0.037 |
| WT1-AS | ZEB1 | hsa-mir-106a | 0.017622 | 0.037 |
| ADAMTS9-AS2 | PPP1R12B | hsa-mir-205 | 0.026156 | 0.036 |
| AGAP11 | AKT3 | hsa-mir-200a | 0.050055 | 0.036 |
| C2orf48 | CXorf36 | hsa-mir-106a | 0.018283 | 0.036 |
| C8orf49 | MASP1 | hsa-mir-143 | 0.127885 | 0.036 |
| CAMK2A | AL713998.1 | hsa-mir-141 | 0.021636 | 0.036 |
| CLRN1-AS1 | ZCCHC24 | hsa-mir-489 | 0.020959 | 0.036 |
| CXCL12 | ADAMTS9-AS2 | hsa-mir-205 | 0.021061 | 0.036 |
| DIO3OS | BNC2 | hsa-mir-143 | 0.133888 | 0.036 |
| HOXC12 | C15orf54 | hsa-mir-195 | 0.020144 | 0.036 |
| KIAA0087 | DPYSL5 | hsa-mir-183 | 0.019667 | 0.036 |
| LINC00314 | RECK | hsa-mir-106a | 0.022066 | 0.036 |
| LINC00355 | MAFA | hsa-mir-122 | 0.022293 | 0.036 |
| LINC00470 | IFFO1 | hsa-mir-424 | 0.09088 | 0.036 |
| MEG3 | TMEM200B | hsa-mir-508 | 0.019453 | 0.036 |
| SFRP4 | LINC00237 | hsa-mir-143 | 0.02178 | 0.036 |
| SLITRK3 | LINC00470 | hsa-mir-200a | 0.024769 | 0.036 |
| WT1-AS | KANK2 | hsa-mir-145 | 0.121953 | 0.036 |
| WT1-AS | GUCY1A2 | hsa-mir-106a | 0.02403 | 0.036 |
| WT1-AS | LPP | hsa-mir-145 | 0.0761 | 0.036 |
| WT1-AS | ASPA | hsa-mir-106a | 0.020331 | 0.036 |
| WT1-AS | TACC1 | hsa-mir-145 | 0.093423 | 0.036 |
| CLDN19 | AGAP11 | hsa-mir-204 | 0.025257 | 0.035 |
| CMAHP | GPRASP1 | hsa-mir-424 | 0.037192 | 0.035 |
| DLX6-AS1 | CACNB2 | hsa-mir-145 | 0.045706 | 0.035 |
| DPYSL5 | WT1-AS | hsa-mir-424 | 0.047146 | 0.035 |
| FLRT2 | ADAMTS9-AS2 | hsa-mir-96 | 0.026083 | 0.035 |
| LMOD1 | AL713998.1 | hsa-mir-141 | 0.021043 | 0.035 |
| MEG3 | MITF | hsa-mir-508 | 0.021483 | 0.035 |
| MRGPRF | LINC00314 | hsa-mir-143 | 0.02669 | 0.035 |
| MUC2 | CENPA | hsa-mir-182 | 0.04877 | 0.035 |
| SYNPO2 | LINC00314 | hsa-mir-143 | 0.02599 | 0.035 |
| T | LINC00355 | hsa-mir-141 | 0.022317 | 0.035 |
| TEK | C20orf166-AS1 | hsa-mir-301b | 0.024292 | 0.035 |
| WT1-AS | KCNMB1 | hsa-mir-363 | 0.020149 | 0.035 |
| ABCG2 | KIAA0087 | hsa-mir-211 | 0.025966 | 0.034 |
| AGAP11 | ANTXR2 | hsa-mir-424 | 0.057438 | 0.034 |
| C11orf96 | LINC00237 | hsa-mir-143 | 0.020037 | 0.034 |
| C2orf48 | KCNJ13 | hsa-mir-143 | 0.021686 | 0.034 |
| CHRNA4 | LINC00355 | hsa-mir-122 | 0.039108 | 0.034 |
| CLRN1-AS1 | KRTAP4-9 | hsa-mir-195 | 0.033508 | 0.034 |
| CXCL12 | AGAP11 | hsa-mir-200a | 0.030164 | 0.034 |
| DLX6-AS1 | PHOX2B | hsa-mir-216b | 0.023588 | 0.034 |
| DPP6 | DIO3OS | hsa-mir-143 | 0.04089 | 0.034 |
| HRH3 | CMAHP | hsa-mir-122 | 0.01746 | 0.034 |
| KIAA0087 | SLC35F1 | hsa-mir-195 | 0.022557 | 0.034 |
| LINC00458 | NXPH3 | hsa-mir-204 | 0.064637 | 0.034 |
| LINC00470 | RBMS3 | hsa-mir-508 | 0.012704 | 0.034 |
| LINC00470 | KANK2 | hsa-mir-424 | 0.05971 | 0.034 |
| LINC00483 | MYLK | hsa-mir-215 | 0.019857 | 0.034 |
| LINC00491 | CSDC2 | hsa-mir-145 | 0.236317 | 0.034 |
| LPP | DIO3OS | hsa-mir-143 | 0.040517 | 0.034 |
| NKX2-1-AS1 | UNC80 | hsa-mir-145 | 0.038851 | 0.034 |
| PENK | C10orf126 | hsa-mir-200a | 0.024917 | 0.034 |
| PLSCR4 | MAGI2-AS3 | hsa-mir-210 | 0.060717 | 0.034 |
| SCGN | KIAA0087 | hsa-mir-195 | 0.029215 | 0.034 |
| AGAP11 | JPH4 | hsa-mir-424 | 0.077431 | 0.033 |
| AKT3 | BX255923.1 | hsa-mir-211 | 0.047666 | 0.033 |
| BPIFA1 | KIAA0087 | hsa-mir-145 | 0.035251 | 0.033 |
| CAMK2A | C20orf166-AS1 | hsa-mir-489 | 0.021995 | 0.033 |
| CMAHP | DGKB | hsa-mir-182 | 0.028026 | 0.033 |
| FLRT2 | LINC00237 | hsa-mir-143 | 0.019402 | 0.033 |
| KANK2 | WT1-AS | hsa-mir-145 | 0.127564 | 0.033 |
| KCNA1 | LINC00523 | hsa-mir-200a | 0.025513 | 0.033 |
| KLF17 | LINC00314 | hsa-mir-211 | 0.043227 | 0.033 |
| LHFP | KIAA0087 | hsa-mir-141 | 0.018624 | 0.033 |
| LINC00337 | NECAB1 | hsa-mir-383 | 0.036056 | 0.033 |
| LINC00473 | KLHL38 | hsa-mir-195 | 0.055591 | 0.033 |
| LINC00483 | SLC16A7 | hsa-mir-215 | 0.014471 | 0.033 |
| LSAMP-AS1 | FAM9C | hsa-mir-183 | 0.016959 | 0.033 |
| OSR1 | MUC2 | hsa-mir-145 | 0.013726 | 0.033 |
| VWC2 | RMST | hsa-mir-195 | 0.023306 | 0.033 |
| WT1-AS | TCF23 | hsa-mir-363 | 0.020104 | 0.033 |
| ABCG2 | DIO3OS | hsa-mir-143 | 0.040265 | 0.032 |
| C20orf166-AS1 | CAMK2A | hsa-mir-489 | 0.023381 | 0.032 |
| CNRIP1 | AC112721.1 | hsa-mir-195 | 0.048649 | 0.032 |
| CNRIP1 | LINC00491 | hsa-mir-145 | 0.018073 | 0.032 |
| FAM41C | DPP6 | hsa-mir-200a | 0.067252 | 0.032 |
| FBXO32 | DIO3OS | hsa-mir-143 | 0.042554 | 0.032 |
| FRMD6-AS2 | HRH3 | hsa-mir-184 | 0.029667 | 0.032 |
| KDR | LINC00211 | hsa-mir-204 | 0.016165 | 0.032 |
| LINC00052 | KLHL4 | hsa-mir-489 | 0.015318 | 0.032 |
| LINC00237 | CAMK2A | hsa-mir-143 | 0.085638 | 0.032 |
| MEIS3 | FAM41C | hsa-mir-200a | 0.036904 | 0.032 |
| MRVI1 | WT1-AS | hsa-mir-363 | 0.026562 | 0.032 |
| MYOCD | DIO3OS | hsa-mir-143 | 0.03869 | 0.032 |
| SLC16A7 | C2orf48 | hsa-mir-424 | 0.017239 | 0.032 |
| WT1-AS | LDB2 | hsa-mir-106a | 0.021679 | 0.032 |
| AQP1 | LINC00491 | hsa-mir-145 | 0.021473 | 0.031 |
| C8orf49 | WFIKKN2 | hsa-mir-429 | 0.028541 | 0.031 |
| CSDC2 | MIR205HG | hsa-mir-215 | 0.027193 | 0.031 |
| ECM2 | C20orf166-AS1 | hsa-mir-489 | 0.020135 | 0.031 |
| KCNMB1 | LINC00473 | hsa-mir-195 | 0.023936 | 0.031 |
| LINC00052 | DDR2 | hsa-mir-489 | 0.017627 | 0.031 |
| LINC00314 | EMCN | hsa-mir-143 | 0.101044 | 0.031 |
| LINC00483 | DUSP27 | hsa-mir-106a | 0.024989 | 0.031 |
| LPP | C2orf48 | hsa-mir-106a | 0.015504 | 0.031 |
| NAV2-AS2 | PPP1R12B | hsa-mir-182 | 0.034132 | 0.031 |
| PPP1R12B | MEG3 | hsa-mir-508 | 0.016564 | 0.031 |
| PTGFR | LINC00211 | hsa-mir-204 | 0.019316 | 0.031 |
| PTPRN2 | DIO3OS | hsa-mir-215 | 0.019485 | 0.031 |
| SVEP1 | C2orf48 | hsa-mir-424 | 0.017352 | 0.031 |
| WT1-AS | IRS4 | hsa-mir-145 | 0.085954 | 0.031 |
| WT1-AS | MYOCD | hsa-mir-363 | 0.020079 | 0.031 |
| ZEB1 | CMAHP | hsa-mir-424 | 0.028298 | 0.031 |
| CDC25C | MUC2 | hsa-mir-182 | 0.026446 | 0.03 |
| CMAHP | FBXL22 | hsa-mir-424 | 0.036953 | 0.03 |
| CMAHP | SUCNR1 | hsa-mir-205 | 0.031806 | 0.03 |
| CNTN4 | NAV2-AS2 | hsa-mir-182 | 0.025066 | 0.03 |
| FAM72D | MIR210HG | hsa-mir-106a | 0.032503 | 0.03 |
| GPRASP1 | CMAHP | hsa-mir-424 | 0.030354 | 0.03 |
| GUCY1A2 | DLX6-AS1 | hsa-mir-211 | 0.018466 | 0.03 |
| HIST1H2AH | DLX6-AS1 | hsa-mir-200a | 0.026413 | 0.03 |
| KCNJ13 | C2orf48 | hsa-mir-143 | 0.021358 | 0.03 |
| KDR | C8orf49 | hsa-mir-143 | 0.030675 | 0.03 |
| KIAA1644 | LINC00470 | hsa-mir-143 | 0.021259 | 0.03 |
| LHX3 | LINC00494 | hsa-mir-182 | 0.037153 | 0.03 |
| MEG3 | PSD | hsa-mir-301b | 0.015241 | 0.03 |
| MYLK | MEG3 | hsa-mir-205 | 0.025934 | 0.03 |
| NKX2-1-AS1 | PCSK2 | hsa-mir-145 | 0.027994 | 0.03 |
| PLN | LINC00355 | hsa-mir-195 | 0.02902 | 0.03 |
| PTPRN2 | MAGI2-AS3 | hsa-mir-195 | 0.025304 | 0.03 |
| RASSF8 | KIAA0087 | hsa-mir-141 | 0.020082 | 0.03 |
| VRTN | LINC00483 | hsa-mir-216b | 0.031355 | 0.03 |
| WT1-AS | DPYSL5 | hsa-mir-424 | 0.026741 | 0.03 |
| WT1-AS | PRLHR | hsa-mir-145 | 0.103693 | 0.03 |
| ADRA1A | LINC00314 | hsa-mir-106a | 0.020386 | 0.029 |
| C2orf48 | LEPR | hsa-mir-106a | 0.044375 | 0.029 |
| CYP2W1 | MEG3 | hsa-mir-96 | 0.033389 | 0.029 |
| DIO3 | C20orf166-AS1 | hsa-mir-489 | 0.021345 | 0.029 |
| FAM9B | KCNQ1DN | hsa-mir-195 | 0.024476 | 0.029 |
| KCNA1 | LINC00470 | hsa-mir-200a | 0.024264 | 0.029 |
| KIAA0087 | EFNA2 | hsa-mir-145 | 0.017497 | 0.029 |
| KLF17 | C8orf49 | hsa-mir-122 | 0.054231 | 0.029 |
| KLHL1 | LINC00211 | hsa-mir-216b | 0.034042 | 0.029 |
| KRTAP4-7 | DNM3OS | hsa-mir-145 | 0.108013 | 0.029 |
| LINC00314 | SELP | hsa-mir-143 | 0.096247 | 0.029 |
| MRVI1 | CMAHP | hsa-mir-424 | 0.029893 | 0.029 |
| NOVA1-AS1 | KCNA1 | hsa-mir-489 | 0.027456 | 0.029 |
| PBX1 | LINC00470 | hsa-mir-429 | 0.022805 | 0.029 |
| PHOX2B | DLX6-AS1 | hsa-mir-216b | 0.015396 | 0.029 |
| RAB23 | WT1-AS | hsa-mir-106a | 0.039808 | 0.029 |
| RBMS3 | LINC00470 | hsa-mir-508 | 0.018485 | 0.029 |
| TCF23 | DLX6-AS1 | hsa-mir-145 | 0.024728 | 0.029 |
| TRPM1 | CLRN1-AS1 | hsa-mir-140 | 0.02999 | 0.029 |
| WT1-AS | PRKG1 | hsa-mir-145 | 0.13733 | 0.029 |
| AGAP11 | GPR17 | hsa-mir-216b | 0.01668 | 0.028 |
| C10orf126 | VRTN | hsa-mir-215 | 0.013239 | 0.028 |
| C2orf48 | NUDT10 | hsa-mir-424 | 0.092385 | 0.028 |
| CLEC14A | C2orf48 | hsa-mir-106a | 0.01748 | 0.028 |
| DIO3OS | LDB2 | hsa-mir-143 | 0.073251 | 0.028 |
| DIO3OS | FBXO32 | hsa-mir-143 | 0.093841 | 0.028 |
| FBXL7 | LINC00491 | hsa-mir-145 | 0.021712 | 0.028 |
| HIST1H2AI | LINC00211 | hsa-mir-183 | 0.019802 | 0.028 |
| KCNA1 | MAGI2-AS3 | hsa-mir-424 | 0.05059 | 0.028 |
| KIAA0087 | HRH3 | hsa-mir-145 | 0.019272 | 0.028 |
| KIAA1644 | WT1-AS | hsa-mir-145 | 0.127821 | 0.028 |
| LINC00237 | AXL | hsa-mir-143 | 0.042509 | 0.028 |
| LINC00470 | MAGI2 | hsa-mir-140 | 0.020657 | 0.028 |
| PRKG1 | LINC00314 | hsa-mir-143 | 0.029248 | 0.028 |
| RMST | DPYSL5 | hsa-mir-204 | 0.017768 | 0.028 |
| S100A7A | BX255923.1 | hsa-mir-363 | 0.018897 | 0.028 |
| WFIKKN2 | DLX6-AS1 | hsa-mir-429 | 0.019254 | 0.028 |
| WT1-AS | GLI2 | hsa-mir-216b | 0.021114 | 0.028 |
| BNC2 | LINC00314 | hsa-mir-143 | 0.030085 | 0.027 |
| BX255923.1 | S100A7A | hsa-mir-363 | 0.019501 | 0.027 |
| C3orf70 | AGAP11 | hsa-mir-424 | 0.038655 | 0.027 |
| C8orf49 | PAX1 | hsa-mir-424 | 0.039977 | 0.027 |
| CLRN1-AS1 | MAGI2 | hsa-mir-489 | 0.020475 | 0.027 |
| CMAHP | PFKFB4 | hsa-mir-145 | 0.048767 | 0.027 |
| CNGA2 | LINC00483 | hsa-mir-216b | 0.035436 | 0.027 |
| DGKB | LINC00488 | hsa-mir-122 | 0.020683 | 0.027 |
| DGKB | LINC00314 | hsa-mir-143 | 0.029862 | 0.027 |
| DIO3OS | TMEM196 | hsa-mir-143 | 0.02438 | 0.027 |
| DLX6-AS1 | RAB23 | hsa-mir-211 | 0.017008 | 0.027 |
| DPYSL5 | ADAMTS9-AS2 | hsa-mir-143 | 0.053681 | 0.027 |
| FRMD6-AS2 | FAM83C | hsa-mir-182 | 0.024653 | 0.027 |
| GLI2 | LINC00483 | hsa-mir-216b | 0.034184 | 0.027 |
| HIC1 | LINC00237 | hsa-mir-143 | 0.019262 | 0.027 |
| KRTAP1-3 | CLRN1-AS1 | hsa-mir-195 | 0.0211 | 0.027 |
| LINC00483 | PRR9 | hsa-mir-96 | 0.022376 | 0.027 |
| LINC00488 | CNN1 | hsa-mir-205 | 0.02878 | 0.027 |
| MEG3 | SULT1E1 | hsa-mir-211 | 0.032068 | 0.027 |
| NRK | LINC00211 | hsa-mir-301b | 0.026934 | 0.027 |
| PHYHIP | C20orf166-AS1 | hsa-mir-489 | 0.020169 | 0.027 |
| PURG | C8orf49 | hsa-mir-424 | 0.052717 | 0.027 |
| TMEM200A | DLX6-AS1 | hsa-mir-211 | 0.017932 | 0.027 |
| TNS1 | KIAA0087 | hsa-mir-211 | 0.028267 | 0.027 |
| XIRP2 | C8orf49 | hsa-mir-195 | 0.029319 | 0.027 |
| ADAMTS9-AS2 | ASPA | hsa-mir-205 | 0.025246 | 0.026 |
| ADAMTS9-AS2 | TGFB1I1 | hsa-mir-96 | 0.037389 | 0.026 |
| C20orf166-AS1 | WT1 | hsa-mir-106a | 0.041084 | 0.026 |
| C8orf49 | JAM3 | hsa-mir-424 | 0.102535 | 0.026 |
| CLSTN2 | MEG3 | hsa-mir-215 | 0.02457 | 0.026 |
| CNTN4 | LINC00052 | hsa-mir-489 | 0.024413 | 0.026 |
| JPH4 | ADAMTS9-AS2 | hsa-mir-205 | 0.026628 | 0.026 |
| KANK2 | LINC00470 | hsa-mir-424 | 0.023082 | 0.026 |
| LINC00211 | HIST1H2AI | hsa-mir-183 | 0.026716 | 0.026 |
| LINC00355 | DPYSL5 | hsa-mir-424 | 0.029429 | 0.026 |
| MEF2C | MAGI2-AS3 | hsa-mir-210 | 0.062113 | 0.026 |
| MUC2 | NKX2-1 | hsa-mir-183 | 0.01832 | 0.026 |
| ZEB2 | JAZF1-AS1 | hsa-mir-205 | 0.028283 | 0.026 |
| ADAMTS9-AS2 | TIMP3 | hsa-mir-200a | 0.061508 | 0.025 |
| AGAP11 | TNFAIP8L3 | hsa-mir-424 | 0.033029 | 0.025 |
| ASCL5 | AL713998.1 | hsa-mir-200a | 0.027227 | 0.025 |
| BRS3 | LINC00470 | hsa-mir-429 | 0.025162 | 0.025 |
| CLRN1-AS1 | TFAP2B | hsa-mir-211 | 0.020904 | 0.025 |
| DSCR4-IT1 | SEZ6L | hsa-mir-211 | 0.035198 | 0.025 |
| EPHA5 | CLRN1-AS1 | hsa-mir-140 | 0.031653 | 0.025 |
| FRMD6 | MUC2 | hsa-mir-106a | 0.019817 | 0.025 |
| IRS4 | LINC00473 | hsa-mir-195 | 0.023052 | 0.025 |
| KIAA0087 | S100A7A | hsa-mir-424 | 0.035838 | 0.025 |
| KLF2 | LINC00237 | hsa-mir-143 | 0.017677 | 0.025 |
| KRTAP4-9 | DNM3OS | hsa-mir-145 | 0.109959 | 0.025 |
| LINC00473 | FBXO40 | hsa-mir-424 | 0.058327 | 0.025 |
| MEG3 | SLC24A3 | hsa-mir-301b | 0.045448 | 0.025 |
| MEG3 | CDON | hsa-mir-96 | 0.033616 | 0.025 |
| MIR210HG | NEUROD1 | hsa-mir-424 | 0.041616 | 0.025 |
| MUC2 | FAM129A | hsa-mir-145 | 0.034233 | 0.025 |
| MYOCD | CMAHP | hsa-mir-424 | 0.030359 | 0.025 |
| NEUROD1 | C8orf49 | hsa-mir-106a | 0.032461 | 0.025 |
| NXPH3 | C8orf49 | hsa-mir-100 | 0.03398 | 0.025 |
| PBX1 | C10orf126 | hsa-mir-200a | 0.028946 | 0.025 |
| PEG3 | RMST | hsa-mir-424 | 0.024013 | 0.025 |
| PPP1R12B | LINC00337 | hsa-mir-383 | 0.016334 | 0.025 |
| PPP1R12B | C8orf49 | hsa-mir-143 | 0.030206 | 0.025 |
| SYNPO2 | AGAP11 | hsa-mir-424 | 0.0373 | 0.025 |
| VRTN | LINC00113 | hsa-mir-145 | 0.024588 | 0.025 |
| CBX7 | LINC00491 | hsa-mir-145 | 0.02286 | 0.024 |
| CCL14 | LINC00237 | hsa-mir-143 | 0.020523 | 0.024 |
| CLRN1-AS1 | EPHA5 | hsa-mir-140 | 0.042273 | 0.024 |
| KCNMB1 | LINC00355 | hsa-mir-195 | 0.031992 | 0.024 |
| LINC00237 | LRRN4CL | hsa-mir-143 | 0.051183 | 0.024 |
| LINC00355 | NKX2-1 | hsa-mir-200a | 0.021795 | 0.024 |
| MAGEA9 | KIAA0087 | hsa-mir-424 | 0.020524 | 0.024 |
| MEG3 | CYP2W1 | hsa-mir-96 | 0.014892 | 0.024 |
| MEG3 | KIAA1644 | hsa-mir-205 | 0.027239 | 0.024 |
| PPP1R12B | DIO3OS | hsa-mir-143 | 0.041619 | 0.024 |
| RMST | PCSK2 | hsa-mir-211 | 0.025269 | 0.024 |
| RTKN2 | MIR210HG | hsa-mir-122 | 0.027029 | 0.024 |
| TCF23 | LINC00355 | hsa-mir-195 | 0.03047 | 0.024 |
| TNS1 | LINC00355 | hsa-mir-195 | 0.031787 | 0.024 |
| VRTN | ATXN8OS | hsa-mir-210 | 0.024958 | 0.024 |
| C10orf126 | BRS3 | hsa-mir-200a | 0.020767 | 0.023 |
| C2orf48 | CLEC14A | hsa-mir-106a | 0.014527 | 0.023 |
| CBX7 | C20orf166-AS1 | hsa-mir-183 | 0.031236 | 0.023 |
| CMAHP | VSTM4 | hsa-mir-195 | 0.058422 | 0.023 |
| DACT3 | LINC00491 | hsa-mir-145 | 0.023191 | 0.023 |
| FAM83C | FRMD6-AS2 | hsa-mir-182 | 0.030927 | 0.023 |
| HCG23 | VSX2 | hsa-mir-145 | 0.027069 | 0.023 |
| HOXC13 | C15orf54 | hsa-mir-195 | 0.025864 | 0.023 |
| HSD17B6 | LINC00314 | hsa-mir-143 | 0.030787 | 0.023 |
| JPH4 | LINC00355 | hsa-mir-195 | 0.032179 | 0.023 |
| KCNA1 | LINC00211 | hsa-mir-301b | 0.028261 | 0.023 |
| KCNJ9 | LINC00458 | hsa-mir-204 | 0.016482 | 0.023 |
| KIAA0087 | CAMK2A | hsa-mir-211 | 0.019565 | 0.023 |
| LINC00314 | CLMP | hsa-mir-143 | 0.129015 | 0.023 |
| MAGI2 | LINC00052 | hsa-mir-489 | 0.025044 | 0.023 |
| MEG3 | TPH1 | hsa-mir-215 | 0.019974 | 0.023 |
| NECAB1 | LINC00337 | hsa-mir-383 | 0.019978 | 0.023 |
| PTGIS | C2orf48 | hsa-mir-106a | 0.019198 | 0.023 |
| ADAMTS9-AS2 | JPH4 | hsa-mir-205 | 0.026429 | 0.022 |
| AGAP11 | PPP1R12B | hsa-mir-200a | 0.046867 | 0.022 |
| AGAP11 | FAM83C | hsa-mir-204 | 0.029934 | 0.022 |
| C2orf48 | NAALAD2 | hsa-mir-424 | 0.023571 | 0.022 |
| CMAHP | TCEAL7 | hsa-mir-424 | 0.074327 | 0.022 |
| CXCL12 | CMAHP | hsa-mir-424 | 0.033621 | 0.022 |
| DUSP27 | LINC00483 | hsa-mir-106a | 0.034753 | 0.022 |
| EMCN | LINC00237 | hsa-mir-143 | 0.022625 | 0.022 |
| HRH3 | FRMD6-AS2 | hsa-mir-184 | 0.021727 | 0.022 |
| KCNMB1 | LINC00314 | hsa-mir-143 | 0.030808 | 0.022 |
| LINC00052 | CNTN4 | hsa-mir-489 | 0.020047 | 0.022 |
| LINC00211 | AKAP2 | hsa-mir-204 | 0.0292 | 0.022 |
| LINC00355 | NOTUM | hsa-mir-140 | 0.033402 | 0.022 |
| LINC00470 | VIPR2 | hsa-mir-424 | 0.12143 | 0.022 |
| MAFA | LINC00355 | hsa-mir-122 | 0.044178 | 0.022 |
| MAMDC2 | LINC00488 | hsa-mir-205 | 0.016848 | 0.022 |
| MLNR | MIR7-3HG | hsa-mir-211 | 0.022916 | 0.022 |
| MRVI1 | C20orf166-AS1 | hsa-mir-489 | 0.022192 | 0.022 |
| MUC2 | PPP1R12B | hsa-mir-140 | 0.076641 | 0.022 |
| PAX1 | C8orf49 | hsa-mir-424 | 0.056094 | 0.022 |
| RECK | C8orf49 | hsa-mir-424 | 0.058982 | 0.022 |
| WFDC5 | C10orf126 | hsa-mir-141 | 0.020636 | 0.022 |
| WT1-AS | TNS1 | hsa-mir-145 | 0.167097 | 0.022 |
| ZCCHC24 | CLRN1-AS1 | hsa-mir-489 | 0.020897 | 0.022 |
| AC112721.1 | PSD | hsa-mir-195 | 0.034298 | 0.021 |
| BX255923.1 | KCTD4 | hsa-mir-204 | 0.02132 | 0.021 |
| BX255923.1 | MAGEA9 | hsa-mir-363 | 0.023013 | 0.021 |
| C1QTNF7 | KIAA0087 | hsa-mir-141 | 0.022234 | 0.021 |
| C2orf48 | MRVI1 | hsa-mir-122 | 0.020212 | 0.021 |
| C8orf49 | BMPER | hsa-mir-424 | 0.063128 | 0.021 |
| CHRDL1 | LINC00314 | hsa-mir-143 | 0.032432 | 0.021 |
| CMAHP | PTGFR | hsa-mir-424 | 0.042032 | 0.021 |
| CNRIP1 | WT1-AS | hsa-mir-145 | 0.132711 | 0.021 |
| DPP6 | FAM41C | hsa-mir-200a | 0.03846 | 0.021 |
| EFNA2 | LINC00483 | hsa-mir-216b | 0.035261 | 0.021 |
| FRMD6-AS2 | LHX3 | hsa-mir-184 | 0.025537 | 0.021 |
| JAZF1-AS1 | CACNB2 | hsa-mir-205 | 0.023632 | 0.021 |
| KIAA0087 | ABRA | hsa-mir-200a | 0.018072 | 0.021 |
| KIAA1644 | DIO3OS | hsa-mir-143 | 0.045131 | 0.021 |
| KRTAP4-9 | C10orf126 | hsa-mir-363 | 0.029148 | 0.021 |
| LINC00314 | DIXDC1 | hsa-mir-143 | 0.060251 | 0.021 |
| LINC00458 | MRGPRF | hsa-mir-122 | 0.025873 | 0.021 |
| MAGI2-AS3 | ABCC9 | hsa-mir-210 | 0.028412 | 0.021 |
| MITF | MEG3 | hsa-mir-508 | 0.019659 | 0.021 |
| PRICKLE2-AS1 | TEK | hsa-mir-141 | 0.036531 | 0.021 |
| PRSS56 | JAZF1-AS1 | hsa-mir-205 | 0.02887 | 0.021 |
| PTPRB | C8orf49 | hsa-mir-143 | 0.031695 | 0.021 |
| RASL12 | LINC00237 | hsa-mir-143 | 0.024026 | 0.021 |
| SLC24A3 | MEG3 | hsa-mir-301b | 0.024147 | 0.021 |
| TNS1 | ADAMTS9-AS2 | hsa-mir-205 | 0.028478 | 0.021 |
| UGT1A10 | LINC00355 | hsa-mir-424 | 0.026263 | 0.021 |
| UNC80 | DLX6-AS1 | hsa-mir-195 | 0.029414 | 0.021 |
| ZEB1 | WT1-AS | hsa-mir-145 | 0.131412 | 0.021 |
| C3orf70 | LINC00314 | hsa-mir-143 | 0.031586 | 0.02 |
| CDON | FRMD6-AS2 | hsa-mir-184 | 0.019284 | 0.02 |
| DIXDC1 | LINC00314 | hsa-mir-143 | 0.032985 | 0.02 |
| FBXL22 | CMAHP | hsa-mir-424 | 0.035022 | 0.02 |
| FLT4 | LINC00470 | hsa-mir-424 | 0.024026 | 0.02 |
| GDF7 | LINC00470 | hsa-mir-508 | 0.020801 | 0.02 |
| GIMAP1 | LINC00491 | hsa-mir-145 | 0.02244 | 0.02 |
| GPR22 | LINC00470 | hsa-mir-143 | 0.025891 | 0.02 |
| LINC00237 | ZCCHC24 | hsa-mir-143 | 0.126032 | 0.02 |
| LINC00237 | TGFB1I1 | hsa-mir-143 | 0.089675 | 0.02 |
| LINC00314 | ADRA1A | hsa-mir-106a | 0.022238 | 0.02 |
| LINC00523 | KCNA1 | hsa-mir-200a | 0.035831 | 0.02 |
| LMOD1 | LINC00211 | hsa-mir-204 | 0.019142 | 0.02 |
| MEG3 | VGLL3 | hsa-mir-215 | 0.024439 | 0.02 |
| PGM5 | WT1-AS | hsa-mir-106a | 0.04403 | 0.02 |
| PRR9 | LINC00483 | hsa-mir-96 | 0.021519 | 0.02 |
| PTGER3 | LINC00355 | hsa-mir-195 | 0.030205 | 0.02 |
| SLC35F1 | MIR205HG | hsa-mir-122 | 0.018934 | 0.02 |
| SUCNR1 | CMAHP | hsa-mir-205 | 0.029275 | 0.02 |
| TMEM200B | C8orf49 | hsa-mir-143 | 0.031403 | 0.02 |
| WT1 | WT1-AS | hsa-mir-96 | 0.039208 | 0.02 |
| WT1-AS | TMEM200B | hsa-mir-106a | 0.02591 | 0.02 |
| ADAMTS9-AS2 | RUNX1T1 | hsa-mir-96 | 0.036368 | 0.019 |
| ANTXR2 | WT1-AS | hsa-mir-363 | 0.028701 | 0.019 |
| AQP1 | ADAMTS9-AS2 | hsa-mir-96 | 0.030582 | 0.019 |
| BEST3 | KIAA0087 | hsa-mir-429 | 0.020865 | 0.019 |
| C20orf166-AS1 | HMCN2 | hsa-mir-489 | 0.034913 | 0.019 |
| C7 | LINC00314 | hsa-mir-143 | 0.030072 | 0.019 |
| CLRN1-AS1 | VGLL3 | hsa-mir-140 | 0.037795 | 0.019 |
| CLRN1-AS1 | KIF26A | hsa-mir-140 | 0.027134 | 0.019 |
| CMAHP | GUCY1A2 | hsa-mir-424 | 0.12415 | 0.019 |
| CNTN4 | AGAP11 | hsa-mir-200a | 0.03234 | 0.019 |
| DSCR10 | ALPI | hsa-mir-424 | 0.025187 | 0.019 |
| FAM9B | LINC00052 | hsa-mir-363 | 0.020396 | 0.019 |
| LINC00052 | MAGI2 | hsa-mir-489 | 0.023566 | 0.019 |
| LINC00314 | PDLIM3 | hsa-mir-143 | 0.231348 | 0.019 |
| LINC00355 | T | hsa-mir-424 | 0.043061 | 0.019 |
| LINC00470 | TNS1 | hsa-mir-143 | 0.068286 | 0.019 |
| LINC00473 | C7 | hsa-mir-195 | 0.055599 | 0.019 |
| LMOD1 | CMAHP | hsa-mir-424 | 0.03413 | 0.019 |
| MAGI2-AS3 | KCTD12 | hsa-mir-210 | 0.071689 | 0.019 |
| MRVI1 | C2orf48 | hsa-mir-122 | 0.026304 | 0.019 |
| MYLK | WT1-AS | hsa-mir-106a | 0.043478 | 0.019 |
| NECAB1 | C2orf48 | hsa-mir-424 | 0.022064 | 0.019 |
| RMRP | BRS3 | hsa-mir-122 | 0.026875 | 0.019 |
| RMST | MYL1 | hsa-mir-508 | 0.019849 | 0.019 |
| TMEM200B | LINC00473 | hsa-mir-195 | 0.027293 | 0.019 |
| WT1-AS | KIAA1644 | hsa-mir-106a | 0.035224 | 0.019 |
| ADH1B | LINC00470 | hsa-mir-424 | 0.026759 | 0.018 |
| C10orf126 | PBX1 | hsa-mir-200a | 0.019155 | 0.018 |
| C10orf126 | KRTAP4-4 | hsa-mir-363 | 0.018096 | 0.018 |
| C10orf126 | PENK | hsa-mir-200a | 0.030143 | 0.018 |
| CLRN1-AS1 | RGS21 | hsa-mir-211 | 0.044332 | 0.018 |
| CMAHP | LEPR | hsa-mir-424 | 0.034242 | 0.018 |
| DDR2 | AGAP11 | hsa-mir-200a | 0.036156 | 0.018 |
| DIO3OS | ABCG2 | hsa-mir-143 | 0.037852 | 0.018 |
| DIXDC1 | C2orf48 | hsa-mir-106a | 0.022408 | 0.018 |
| DPYSL5 | NAV2-AS2 | hsa-mir-96 | 0.019829 | 0.018 |
| FAM41C | RAB23 | hsa-mir-145 | 0.036994 | 0.018 |
| GPRASP1 | AL713998.1 | hsa-mir-141 | 0.027354 | 0.018 |
| GPRASP1 | RMST | hsa-mir-195 | 0.022617 | 0.018 |
| KIAA0087 | ALPP | hsa-mir-96 | 0.026544 | 0.018 |
| KLHL4 | MAGI2-AS3 | hsa-mir-210 | 0.063124 | 0.018 |
| LDB2 | DIO3OS | hsa-mir-143 | 0.04387 | 0.018 |
| LINC00052 | AKT3 | hsa-mir-489 | 0.02024 | 0.018 |
| LINC00355 | T | hsa-mir-141 | 0.027525 | 0.018 |
| MASP1 | LINC00211 | hsa-mir-204 | 0.019939 | 0.018 |
| MEF2C | DIO3OS | hsa-mir-143 | 0.043508 | 0.018 |
| MITF | C15orf54 | hsa-mir-301b | 0.016468 | 0.018 |
| NKX2-1-AS1 | ARHGEF15 | hsa-mir-211 | 0.032285 | 0.018 |
| OLFML1 | MAGI2-AS3 | hsa-mir-210 | 0.066427 | 0.018 |
| OPCML | LINC00523 | hsa-mir-200a | 0.030696 | 0.018 |
| RMRP | KRTAP4-7 | hsa-mir-122 | 0.06522 | 0.018 |
| TMPRSS11A | KIAA0087 | hsa-mir-96 | 0.020286 | 0.018 |
| TNS1 | WT1-AS | hsa-mir-363 | 0.026384 | 0.018 |
| VSX2 | ATXN8OS | hsa-mir-122 | 0.055947 | 0.018 |
| WT1-AS | MRGPRF | hsa-mir-363 | 0.024834 | 0.018 |
| WT1-AS | ANTXR2 | hsa-mir-363 | 0.023639 | 0.018 |
| ABRA | KIAA0087 | hsa-mir-200a | 0.020814 | 0.017 |
| BRS3 | C10orf126 | hsa-mir-200a | 0.028418 | 0.017 |
| C10orf126 | WFDC5 | hsa-mir-141 | 0.018712 | 0.017 |
| C15orf54 | HOXC13 | hsa-mir-195 | 0.041712 | 0.017 |
| C2orf48 | TACC1 | hsa-mir-106a | 0.025379 | 0.017 |
| CNN1 | LINC00488 | hsa-mir-205 | 0.017219 | 0.017 |
| CPED1 | MAGI2-AS3 | hsa-mir-210 | 0.064091 | 0.017 |
| DIO3OS | ANTXR2 | hsa-mir-143 | 0.077927 | 0.017 |
| DLL3 | GLIS3-AS1 | hsa-mir-100 | 0.023329 | 0.017 |
| HIST1H3I | LINC00211 | hsa-mir-183 | 0.019954 | 0.017 |
| LEPR | C2orf48 | hsa-mir-106a | 0.020872 | 0.017 |
| LINC00483 | GLI2 | hsa-mir-216b | 0.023493 | 0.017 |
| MAGI2-AS3 | SYNPO2 | hsa-mir-210 | 0.0475 | 0.017 |
| MAMDC2 | LINC00211 | hsa-mir-204 | 0.023119 | 0.017 |
| MEG3 | MYOCD | hsa-mir-205 | 0.025533 | 0.017 |
| OLIG3 | CLRN1-AS1 | hsa-mir-424 | 0.051837 | 0.017 |
| PRKG1 | C15orf54 | hsa-mir-301b | 0.018453 | 0.017 |
| PTGER3 | DIO3OS | hsa-mir-143 | 0.044806 | 0.017 |
| RECK | LINC00314 | hsa-mir-106a | 0.023571 | 0.017 |
| RUNX1T1 | LINC00237 | hsa-mir-143 | 0.024349 | 0.017 |
| SELP | LINC00473 | hsa-mir-195 | 0.027572 | 0.017 |
| ADTRP | MAGI2-AS3 | hsa-mir-143 | 0.097444 | 0.016 |
| AKT3 | MEG3 | hsa-mir-205 | 0.030326 | 0.016 |
| AKT3 | LINC00052 | hsa-mir-489 | 0.026175 | 0.016 |
| CHRNA4 | AGAP11 | hsa-mir-204 | 0.031407 | 0.016 |
| DACT3 | LINC00237 | hsa-mir-143 | 0.024859 | 0.016 |
| DDR2 | MAGI2-AS3 | hsa-mir-210 | 0.063354 | 0.016 |
| DGKB | LINC00355 | hsa-mir-141 | 0.025966 | 0.016 |
| DLX6-AS1 | UNC80 | hsa-mir-195 | 0.0608 | 0.016 |
| EFNA2 | DSCR4-IT1 | hsa-mir-508 | 0.021693 | 0.016 |
| GUCY1A2 | WT1-AS | hsa-mir-106a | 0.044597 | 0.016 |
| KANK2 | LINC00237 | hsa-mir-143 | 0.023836 | 0.016 |
| KLHDC8A | CMAHP | hsa-mir-195 | 0.032761 | 0.016 |
| LINC00211 | PBOV1 | hsa-mir-183 | 0.032656 | 0.016 |
| LINC00211 | MYOG | hsa-mir-301b | 0.025334 | 0.016 |
| LINC00458 | AMER2 | hsa-mir-143 | 0.032865 | 0.016 |
| LINC00473 | DGKB | hsa-mir-195 | 0.035191 | 0.016 |
| LMOD1 | DIO3OS | hsa-mir-143 | 0.04778 | 0.016 |
| MASP1 | WT1-AS | hsa-mir-363 | 0.029589 | 0.016 |
| MEG3 | TMEM200A | hsa-mir-508 | 0.028161 | 0.016 |
| MEG3 | SYT1 | hsa-mir-215 | 0.028903 | 0.016 |
| MEG3 | ZFPM2 | hsa-mir-508 | 0.035043 | 0.016 |
| MEG3 | NRK | hsa-mir-211 | 0.024211 | 0.016 |
| MUC2 | CDC25C | hsa-mir-182 | 0.037609 | 0.016 |
| MYOCD | MEG3 | hsa-mir-301b | 0.027798 | 0.016 |
| NKX2-1-AS1 | T | hsa-mir-145 | 0.043137 | 0.016 |
| RMRP | KRTAP3-2 | hsa-mir-122 | 0.057593 | 0.016 |
| T | LINC00458 | hsa-mir-205 | 0.033408 | 0.016 |
| TAL1 | FAM41C | hsa-mir-200a | 0.040503 | 0.016 |
| UGT1A8 | LINC00355 | hsa-mir-424 | 0.02691 | 0.016 |
| WT1-AS | WT1 | hsa-mir-96 | 0.046268 | 0.016 |
| ZNF366 | LINC00211 | hsa-mir-204 | 0.022561 | 0.016 |
| ADAMTS9-AS2 | RSPO1 | hsa-mir-96 | 0.040231 | 0.015 |
| AGAP11 | PLN | hsa-mir-424 | 0.046385 | 0.015 |
| AKT3 | MEG3 | hsa-mir-508 | 0.022542 | 0.015 |
| AKT3 | AGAP11 | hsa-mir-200a | 0.039065 | 0.015 |
| C10orf126 | TPTE | hsa-mir-508 | 0.021161 | 0.015 |
| C20orf166-AS1 | C1QTNF7 | hsa-mir-489 | 0.024222 | 0.015 |
| CMAHP | DDC | hsa-mir-182 | 0.028262 | 0.015 |
| GPR12 | CLRN1-AS1 | hsa-mir-211 | 0.020438 | 0.015 |
| GPRASP1 | WT1-AS | hsa-mir-363 | 0.02915 | 0.015 |
| HSD17B13 | RMST | hsa-mir-211 | 0.035993 | 0.015 |
| IGF2BP3 | C8orf49 | hsa-mir-195 | 0.031448 | 0.015 |
| KIAA1462 | CMAHP | hsa-mir-424 | 0.036397 | 0.015 |
| LINC00052 | NAALAD2 | hsa-mir-489 | 0.019219 | 0.015 |
| LINC00355 | KCNMB1 | hsa-mir-195 | 0.053941 | 0.015 |
| LINC00443 | VRTN | hsa-mir-363 | 0.022239 | 0.015 |
| LINC00494 | LHX3 | hsa-mir-182 | 0.021893 | 0.015 |
| MAGEA9 | BX255923.1 | hsa-mir-363 | 0.024101 | 0.015 |
| MASP1 | LINC00473 | hsa-mir-195 | 0.025173 | 0.015 |
| MRVI1 | LINC00314 | hsa-mir-143 | 0.03189 | 0.015 |
| MUC2 | SOX7 | hsa-mir-106a | 0.025569 | 0.015 |
| MYOG | LINC00470 | hsa-mir-200a | 0.029541 | 0.015 |
| OGN | LINC00211 | hsa-mir-204 | 0.022769 | 0.015 |
| POU6F2-AS2 | SCGN | hsa-mir-383 | 0.037352 | 0.015 |
| PRKG1 | MAGI2-AS3 | hsa-mir-210 | 0.068114 | 0.015 |
| RMST | DPYSL5 | hsa-mir-211 | 0.022869 | 0.015 |
| RUNX1T1 | ADAMTS9-AS2 | hsa-mir-96 | 0.031221 | 0.015 |
| SALL3 | LINC00458 | hsa-mir-143 | 0.036182 | 0.015 |
| TPTE | LINC00470 | hsa-mir-200a | 0.028713 | 0.015 |
| YPEL4 | ADAMTS9-AS2 | hsa-mir-96 | 0.030644 | 0.015 |
| AGTR1 | CLRN1-AS1 | hsa-mir-211 | 0.018569 | 0.014 |
| ANTXR2 | DIO3OS | hsa-mir-143 | 0.044526 | 0.014 |
| ATXN8OS | VRTN | hsa-mir-210 | 0.022797 | 0.014 |
| BRS3 | LINC00051 | hsa-mir-205 | 0.031253 | 0.014 |
| CLRN1-AS1 | CLMP | hsa-mir-489 | 0.021705 | 0.014 |
| DGKB | NKX2-1-AS1 | hsa-mir-145 | 0.044379 | 0.014 |
| DIO3OS | C3orf70 | hsa-mir-143 | 0.158699 | 0.014 |
| DLX6-AS1 | WFIKKN2 | hsa-mir-429 | 0.033133 | 0.014 |
| DNM3OS | KRT33B | hsa-mir-145 | 0.042222 | 0.014 |
| GRID1 | LINC00470 | hsa-mir-424 | 0.027022 | 0.014 |
| HRH3 | NKX2-1-AS1 | hsa-mir-145 | 0.044725 | 0.014 |
| KLHL38 | LINC00355 | hsa-mir-195 | 0.033184 | 0.014 |
| KRTAP4-9 | CLRN1-AS1 | hsa-mir-195 | 0.021543 | 0.014 |
| LINC00052 | VGLL3 | hsa-mir-489 | 0.016763 | 0.014 |
| LINC00211 | TCF23 | hsa-mir-204 | 0.060905 | 0.014 |
| LINC00393 | IRS4 | hsa-mir-106a | 0.033274 | 0.014 |
| LINC00470 | BRS3 | hsa-mir-429 | 0.019483 | 0.014 |
| LMOD1 | WT1-AS | hsa-mir-106a | 0.042276 | 0.014 |
| MAGI2-AS3 | GPR12 | hsa-mir-143 | 0.031835 | 0.014 |
| MEG3 | PLN | hsa-mir-205 | 0.028599 | 0.014 |
| RFX6 | LINC00052 | hsa-mir-425 | 0.021145 | 0.014 |
| TCF23 | LINC00211 | hsa-mir-204 | 0.021185 | 0.014 |
| TEK | PRICKLE2-AS1 | hsa-mir-141 | 0.021912 | 0.014 |
| WT1-AS | RAB23 | hsa-mir-106a | 0.020792 | 0.014 |
| ABCC9 | RMST | hsa-mir-195 | 0.027124 | 0.013 |
| AGAP11 | MSRB3 | hsa-mir-424 | 0.063267 | 0.013 |
| BMPER | C8orf49 | hsa-mir-424 | 0.061037 | 0.013 |
| C10orf126 | GPR83 | hsa-mir-140 | 0.025619 | 0.013 |
| CACNB2 | JAZF1-AS1 | hsa-mir-205 | 0.032176 | 0.013 |
| CAMK2A | LINC00355 | hsa-mir-195 | 0.032351 | 0.013 |
| CXorf36 | C2orf48 | hsa-mir-106a | 0.022024 | 0.013 |
| DIO3OS | PTPRN2 | hsa-mir-215 | 0.030561 | 0.013 |
| DLX6-AS1 | DPYSL5 | hsa-mir-195 | 0.044672 | 0.013 |
| FAM129A | MUC2 | hsa-mir-145 | 0.020679 | 0.013 |
| FAM41C | TNS1 | hsa-mir-200a | 0.071941 | 0.013 |
| FBXL22 | LINC00470 | hsa-mir-143 | 0.028679 | 0.013 |
| FBXO40 | C8orf49 | hsa-mir-122 | 0.058245 | 0.013 |
| LINC00355 | VRTN | hsa-mir-122 | 0.042906 | 0.013 |
| LINC00488 | NEXN | hsa-mir-205 | 0.042284 | 0.013 |
| MAGI2-AS3 | MCM10 | hsa-mir-143 | 0.033746 | 0.013 |
| MEG3 | SLITRK3 | hsa-mir-211 | 0.048537 | 0.013 |
| MEG3 | TAL1 | hsa-mir-508 | 0.029695 | 0.013 |
| NAALAD2 | C2orf48 | hsa-mir-424 | 0.02393 | 0.013 |
| NAV3 | LINC00523 | hsa-mir-200a | 0.035132 | 0.013 |
| NOVA1-AS1 | ADRB3 | hsa-mir-489 | 0.025009 | 0.013 |
| PDLIM3 | DIO3OS | hsa-mir-143 | 0.048853 | 0.013 |
| SYNPO2 | LINC00470 | hsa-mir-143 | 0.029772 | 0.013 |
| TAL1 | LINC00314 | hsa-mir-143 | 0.032774 | 0.013 |
| TMEM132D | CLRN1-AS1 | hsa-mir-140 | 0.033479 | 0.013 |
| TMEM196 | LINC00314 | hsa-mir-143 | 0.032174 | 0.013 |
| TMEM200B | MEG3 | hsa-mir-508 | 0.020641 | 0.013 |
| TMPRSS11A | LINC00491 | hsa-mir-122 | 0.035471 | 0.013 |
| WT1-AS | MRVI1 | hsa-mir-363 | 0.02638 | 0.013 |
| WT1-AS | GPRASP1 | hsa-mir-363 | 0.022393 | 0.013 |
| WT1-AS | GPRASP1 | hsa-mir-106a | 0.023088 | 0.013 |
| AGAP11 | CLDN19 | hsa-mir-204 | 0.03246 | 0.012 |
| BNC2 | DIO3OS | hsa-mir-143 | 0.045076 | 0.012 |
| BPIFA1 | LINC00483 | hsa-mir-216b | 0.041367 | 0.012 |
| C7 | LINC00473 | hsa-mir-195 | 0.028028 | 0.012 |
| CHRDL1 | DIO3OS | hsa-mir-143 | 0.046947 | 0.012 |
| CNGA2 | LINC00355 | hsa-mir-122 | 0.051657 | 0.012 |
| CPED1 | WT1-AS | hsa-mir-106a | 0.046836 | 0.012 |
| DPYSL5 | DLX6-AS1 | hsa-mir-195 | 0.037342 | 0.012 |
| FAM41C | TAL1 | hsa-mir-200a | 0.049768 | 0.012 |
| FLT4 | LINC00237 | hsa-mir-143 | 0.024591 | 0.012 |
| GUCY1A2 | NAV2-AS2 | hsa-mir-182 | 0.031475 | 0.012 |
| LINC00211 | ZNF366 | hsa-mir-204 | 0.045004 | 0.012 |
| LINC00355 | KLHL38 | hsa-mir-195 | 0.05887 | 0.012 |
| LINC00483 | CNGA2 | hsa-mir-216b | 0.045465 | 0.012 |
| MAGI2-AS3 | VGLL3 | hsa-mir-210 | 0.035982 | 0.012 |
| MAMDC2 | LINC00470 | hsa-mir-143 | 0.027849 | 0.012 |
| MEG3 | MYLK | hsa-mir-205 | 0.023834 | 0.012 |
| MIR210HG | FAM72D | hsa-mir-106a | 0.023034 | 0.012 |
| MMRN2 | DIO3OS | hsa-mir-508 | 0.022585 | 0.012 |
| MSRB3 | KIAA0087 | hsa-mir-141 | 0.023163 | 0.012 |
| NAV2-AS2 | SYNPO2 | hsa-mir-182 | 0.026709 | 0.012 |
| NRK | MEG3 | hsa-mir-211 | 0.03411 | 0.012 |
| OLFML1 | C2orf48 | hsa-mir-424 | 0.024156 | 0.012 |
| PLN | LINC00488 | hsa-mir-205 | 0.018163 | 0.012 |
| RERG | LINC00211 | hsa-mir-204 | 0.018713 | 0.012 |
| RSPO3 | RMST | hsa-mir-301b | 0.032362 | 0.012 |
| SCGN | POU6F2-AS2 | hsa-mir-383 | 0.040002 | 0.012 |
| SLC14A2 | CMAHP | hsa-mir-195 | 0.037723 | 0.012 |
| TROAP | AC107959.1 | hsa-mir-301b | 0.018436 | 0.012 |
| VSX2 | NKX2-1-AS1 | hsa-mir-96 | 0.027763 | 0.012 |
| WT1-AS | GPR22 | hsa-mir-145 | 0.113897 | 0.012 |
| WT1-AS | SYNPO2 | hsa-mir-106a | 0.022119 | 0.012 |
| WT1-AS | MRVI1 | hsa-mir-106a | 0.023702 | 0.012 |
| AGAP11 | TSHZ3 | hsa-mir-200a | 0.091681 | 0.011 |
| AL713998.1 | ASCL5 | hsa-mir-200a | 0.032987 | 0.011 |
| C20orf166-AS1 | LMOD1 | hsa-mir-489 | 0.02426 | 0.011 |
| C2orf48 | MSRB3 | hsa-mir-424 | 0.066686 | 0.011 |
| C8orf49 | RECK | hsa-mir-424 | 0.046201 | 0.011 |
| CLEC14A | WT1-AS | hsa-mir-145 | 0.140583 | 0.011 |
| DIO3OS | TMEM200B | hsa-mir-143 | 0.149248 | 0.011 |
| ECM2 | LINC00237 | hsa-mir-143 | 0.027369 | 0.011 |
| FIBCD1 | ADARB2-AS1 | hsa-mir-205 | 0.044594 | 0.011 |
| JAZF1-AS1 | CLSTN2 | hsa-mir-205 | 0.029409 | 0.011 |
| KLF2 | WT1-AS | hsa-mir-145 | 0.142412 | 0.011 |
| KRTAP4-7 | RMRP | hsa-mir-122 | 0.057946 | 0.011 |
| LINC00355 | MYOCD | hsa-mir-195 | 0.114553 | 0.011 |
| LINC00458 | T | hsa-mir-205 | 0.035368 | 0.011 |
| LINC00458 | BTBD17 | hsa-mir-200a | 0.055176 | 0.011 |
| LINC00470 | NR3C1 | hsa-mir-508 | 0.036695 | 0.011 |
| LMOD1 | LINC00355 | hsa-mir-195 | 0.035247 | 0.011 |
| MAGI2-AS3 | EDNRA | hsa-mir-210 | 0.050836 | 0.011 |
| MAGI2-AS3 | CPED1 | hsa-mir-210 | 0.058307 | 0.011 |
| MEG3 | AKT3 | hsa-mir-205 | 0.024701 | 0.011 |
| MMRN2 | CMAHP | hsa-mir-122 | 0.021665 | 0.011 |
| MYOCD | LINC00470 | hsa-mir-143 | 0.031392 | 0.011 |
| NECAB1 | NOVA1-AS1 | hsa-mir-425 | 0.03087 | 0.011 |
| OLIG3 | ATXN8OS | hsa-mir-183 | 0.029075 | 0.011 |
| PPP1R12B | AGAP11 | hsa-mir-200a | 0.038134 | 0.011 |
| PTGIS | LINC00314 | hsa-mir-143 | 0.035003 | 0.011 |
| RECK | KIAA0087 | hsa-mir-141 | 0.023464 | 0.011 |
| RMST | PPP1R12B | hsa-mir-195 | 0.110409 | 0.011 |
| SEZ6L | C8orf49 | hsa-mir-100 | 0.042164 | 0.011 |
| SEZ6L | CMAHP | hsa-mir-141 | 0.04577 | 0.011 |
| TNS1 | FAM41C | hsa-mir-200a | 0.048673 | 0.011 |
| WT1-AS | MYCT1 | hsa-mir-106a | 0.035634 | 0.011 |
| WT1-AS | MEF2C | hsa-mir-145 | 0.164812 | 0.011 |
| AGTR1 | DIO3OS | hsa-mir-211 | 0.020359 | 0.01 |
| ASPA | MEG3 | hsa-mir-205 | 0.032751 | 0.01 |
| ATXN8OS | EFNA2 | hsa-mir-211 | 0.030358 | 0.01 |
| C8orf49 | SMIM10 | hsa-mir-424 | 0.040627 | 0.01 |
| CXorf36 | C20orf166-AS1 | hsa-mir-183 | 0.036761 | 0.01 |
| DIO3OS | GRK5 | hsa-mir-211 | 0.028678 | 0.01 |
| DNM3OS | KRTAP4-9 | hsa-mir-145 | 0.045577 | 0.01 |
| FBXO32 | WT1-AS | hsa-mir-145 | 0.144587 | 0.01 |
| HIC1 | LINC00470 | hsa-mir-424 | 0.031042 | 0.01 |
| KIAA0087 | TNS1 | hsa-mir-211 | 0.023416 | 0.01 |
| KIAA1644 | WT1-AS | hsa-mir-106a | 0.04818 | 0.01 |
| LINC00051 | KRTAP4-4 | hsa-mir-205 | 0.023813 | 0.01 |
| LINC00052 | RFX6 | hsa-mir-425 | 0.023563 | 0.01 |
| LINC00052 | FAM9B | hsa-mir-363 | 0.024867 | 0.01 |
| LINC00211 | KCNA1 | hsa-mir-301b | 0.027856 | 0.01 |
| LINC00211 | IRS4 | hsa-mir-204 | 0.092877 | 0.01 |
| LINC00483 | VRTN | hsa-mir-216b | 0.028542 | 0.01 |
| LINC00488 | STXBP5L | hsa-mir-216b | 0.038645 | 0.01 |
| MAGI2-AS3 | PLSCR4 | hsa-mir-210 | 0.045087 | 0.01 |
| MEG3 | KLHL4 | hsa-mir-508 | 0.032156 | 0.01 |
| MRGPRF | LINC00470 | hsa-mir-424 | 0.026672 | 0.01 |
| NAALAD2 | LINC00337 | hsa-mir-383 | 0.024349 | 0.01 |
| NOVA1-AS1 | SLITRK3 | hsa-mir-489 | 0.026227 | 0.01 |
| POU6F2-AS2 | PCSK2 | hsa-mir-383 | 0.025389 | 0.01 |
| RASL12 | LINC00470 | hsa-mir-424 | 0.027675 | 0.01 |
| RMST | HSD17B13 | hsa-mir-211 | 0.027585 | 0.01 |
| SNCA | MAGI2-AS3 | hsa-mir-143 | 0.099751 | 0.01 |
| SYNPO2 | MEG3 | hsa-mir-205 | 0.03174 | 0.01 |
| TACC1 | C2orf48 | hsa-mir-106a | 0.020859 | 0.01 |
| TCEAL6 | DIO3OS | hsa-mir-143 | 0.048262 | 0.01 |
| VRTN | LINC00443 | hsa-mir-363 | 0.028162 | 0.01 |
| WT1-AS | CALD1 | hsa-mir-363 | 0.02312 | 0.01 |
| WT1-AS | CPED1 | hsa-mir-106a | 0.022369 | 0.01 |
| WT1-AS | STXBP5L | hsa-mir-424 | 0.024586 | 0.01 |
| ZCCHC24 | LINC00052 | hsa-mir-489 | 0.031422 | 0.01 |
| ADAMTS9-AS2 | DPYSL5 | hsa-mir-143 | 0.029963 | 0.009 |
| ANTXR2 | WT1-AS | hsa-mir-145 | 0.142173 | 0.009 |
| ASPN | DIO3OS | hsa-mir-143 | 0.049952 | 0.009 |
| AXL | LINC00237 | hsa-mir-143 | 0.0351 | 0.009 |
| C10orf126 | SEZ6L | hsa-mir-140 | 0.023525 | 0.009 |
| C20orf166-AS1 | DIO3 | hsa-mir-489 | 0.03128 | 0.009 |
| C20orf166-AS1 | DIXDC1 | hsa-mir-183 | 0.028814 | 0.009 |
| C8orf49 | PLN | hsa-mir-143 | 0.280875 | 0.009 |
| C8orf49 | CDON | hsa-mir-429 | 0.042704 | 0.009 |
| CALD1 | WT1-AS | hsa-mir-363 | 0.03479 | 0.009 |
| DACT3 | DIO3OS | hsa-mir-143 | 0.046647 | 0.009 |
| EMX1 | LINC00458 | hsa-mir-143 | 0.038556 | 0.009 |
| FAM9C | ADARB2-AS1 | hsa-mir-205 | 0.049382 | 0.009 |
| GUCY1A2 | C20orf166-AS1 | hsa-mir-183 | 0.036445 | 0.009 |
| KCNA1 | NOVA1-AS1 | hsa-mir-489 | 0.032356 | 0.009 |
| KIAA1644 | LINC00355 | hsa-mir-195 | 0.041316 | 0.009 |
| KLF17 | DLX6-AS1 | hsa-mir-145 | 0.032848 | 0.009 |
| KLHDC8A | WT1-AS | hsa-mir-383 | 0.062475 | 0.009 |
| KLHL4 | LINC00052 | hsa-mir-489 | 0.026222 | 0.009 |
| KLHL4 | MEG3 | hsa-mir-508 | 0.024415 | 0.009 |
| KRTAP4-6 | CLRN1-AS1 | hsa-mir-195 | 0.030739 | 0.009 |
| LINC00052 | ZCCHC24 | hsa-mir-489 | 0.029576 | 0.009 |
| LINC00211 | GPR52 | hsa-mir-183 | 0.043129 | 0.009 |
| LINC00211 | SPHKAP | hsa-mir-301b | 0.031017 | 0.009 |
| LINC00314 | ABRA | hsa-mir-211 | 0.022193 | 0.009 |
| LINC00470 | FOXL2 | hsa-mir-424 | 0.191844 | 0.009 |
| LINC00491 | TMPRSS11A | hsa-mir-122 | 0.068128 | 0.009 |
| MAGI2-AS3 | SNCA | hsa-mir-143 | 0.040998 | 0.009 |
| MAGI2-AS3 | PPP1R12B | hsa-mir-210 | 0.039968 | 0.009 |
| MYL1 | RMST | hsa-mir-508 | 0.028404 | 0.009 |
| MYOCD | C8orf49 | hsa-mir-143 | 0.037632 | 0.009 |
| NAV2-AS2 | LHX3 | hsa-mir-204 | 0.022952 | 0.009 |
| NUDT10 | C2orf48 | hsa-mir-424 | 0.027774 | 0.009 |
| OR5C1 | LINC00314 | hsa-mir-211 | 0.052825 | 0.009 |
| PAX1 | LINC00470 | hsa-mir-508 | 0.026619 | 0.009 |
| PHOX2B | NKX2-1-AS1 | hsa-mir-301b | 0.037084 | 0.009 |
| RTKN2 | LINC00052 | hsa-mir-216b | 0.054209 | 0.009 |
| SCRG1 | NKX2-1-AS1 | hsa-mir-145 | 0.047614 | 0.009 |
| SPHKAP | LINC00211 | hsa-mir-301b | 0.033306 | 0.009 |
| SYTL4 | AC092422.1 | hsa-mir-424 | 0.029689 | 0.009 |
| TCF23 | CMAHP | hsa-mir-424 | 0.036133 | 0.009 |
| TMEM200B | LINC00470 | hsa-mir-143 | 0.030473 | 0.009 |
| TPTE | C10orf126 | hsa-mir-508 | 0.032947 | 0.009 |
| ZNF540 | ADAMTS9-AS2 | hsa-mir-96 | 0.036717 | 0.009 |
| ABCG2 | LINC00211 | hsa-mir-204 | 0.026098 | 0.008 |
| AKAP2 | LINC00211 | hsa-mir-204 | 0.024903 | 0.008 |
| ASPA | C8orf49 | hsa-mir-143 | 0.044635 | 0.008 |
| ATXN8OS | VSX2 | hsa-mir-122 | 0.03598 | 0.008 |
| ATXN8OS | WFIKKN2 | hsa-mir-211 | 0.026013 | 0.008 |
| ATXN8OS | OLIG3 | hsa-mir-183 | 0.028414 | 0.008 |
| C2orf48 | KIAA1462 | hsa-mir-106a | 0.027628 | 0.008 |
| C2orf48 | PTGIS | hsa-mir-106a | 0.030859 | 0.008 |
| C2orf48 | DIXDC1 | hsa-mir-106a | 0.037331 | 0.008 |
| CDON | MEG3 | hsa-mir-96 | 0.047327 | 0.008 |
| CENPA | MUC2 | hsa-mir-182 | 0.032511 | 0.008 |
| CFHR4 | FRMD6-AS2 | hsa-mir-96 | 0.030423 | 0.008 |
| CLMP | C2orf48 | hsa-mir-424 | 0.023725 | 0.008 |
| CLRN1-AS1 | MITF | hsa-mir-140 | 0.045436 | 0.008 |
| CLSTN2 | JAZF1-AS1 | hsa-mir-205 | 0.03598 | 0.008 |
| CMAHP | CALML3 | hsa-mir-141 | 0.03436 | 0.008 |
| DDR2 | KIAA0087 | hsa-mir-141 | 0.02999 | 0.008 |
| DSCR10 | TMEM151A | hsa-mir-424 | 0.031719 | 0.008 |
| HOXC13 | LINC00052 | hsa-mir-425 | 0.026153 | 0.008 |
| IRS4 | LINC00393 | hsa-mir-106a | 0.034838 | 0.008 |
| JAZF1-AS1 | PRSS56 | hsa-mir-205 | 0.03833 | 0.008 |
| JAZF1-AS1 | ZEB2 | hsa-mir-205 | 0.025684 | 0.008 |
| JPH4 | AGAP11 | hsa-mir-424 | 0.05121 | 0.008 |
| KIAA0087 | BEST3 | hsa-mir-429 | 0.02114 | 0.008 |
| KLF17 | MAGI2-AS3 | hsa-mir-425 | 0.037262 | 0.008 |
| KRTAP4-4 | LINC00051 | hsa-mir-205 | 0.034281 | 0.008 |
| LDB2 | WT1-AS | hsa-mir-106a | 0.0483 | 0.008 |
| LHX3 | FRMD6-AS2 | hsa-mir-182 | 0.038711 | 0.008 |
| LHX3 | ATXN8OS | hsa-mir-211 | 0.065043 | 0.008 |
| LINC00237 | ROBO4 | hsa-mir-143 | 0.063697 | 0.008 |
| LINC00355 | LMOD1 | hsa-mir-195 | 0.083495 | 0.008 |
| LINC00470 | AXL | hsa-mir-424 | 0.106464 | 0.008 |
| MAGI2-AS3 | AKT3 | hsa-mir-210 | 0.052976 | 0.008 |
| MEG3 | PPP1R12B | hsa-mir-205 | 0.034784 | 0.008 |
| MRVI1 | LINC00470 | hsa-mir-424 | 0.030398 | 0.008 |
| NOVA1-AS1 | POU6F2 | hsa-mir-489 | 0.040567 | 0.008 |
| RSPO1 | LINC00314 | hsa-mir-143 | 0.043065 | 0.008 |
| SCGN | RMST | hsa-mir-211 | 0.040614 | 0.008 |
| SOX7 | MUC2 | hsa-mir-106a | 0.027361 | 0.008 |
| TAL1 | LINC00491 | hsa-mir-145 | 0.032651 | 0.008 |
| TMEM200A | WT1-AS | hsa-mir-106a | 0.052401 | 0.008 |
| TMEM200B | KIAA0087 | hsa-mir-141 | 0.026333 | 0.008 |
| VGLL3 | MAGI2-AS3 | hsa-mir-210 | 0.068939 | 0.008 |
| WT1-AS | KLF2 | hsa-mir-145 | 0.120032 | 0.008 |
| WT1-AS | MYLK | hsa-mir-106a | 0.032897 | 0.008 |
| ZEB1 | C2orf48 | hsa-mir-424 | 0.025623 | 0.008 |
| AGAP11 | GUCA2A | hsa-mir-195 | 0.036672 | 0.007 |
| CAMK2A | LINC00237 | hsa-mir-143 | 0.028105 | 0.007 |
| CPED1 | DIO3OS | hsa-mir-143 | 0.05237 | 0.007 |
| DLX3 | LINC00458 | hsa-mir-204 | 0.024499 | 0.007 |
| DLX6-AS1 | GUCY1A2 | hsa-mir-211 | 0.025961 | 0.007 |
| DLX6-AS1 | MYH11 | hsa-mir-211 | 0.036419 | 0.007 |
| DNM3OS | KRTAP4-7 | hsa-mir-145 | 0.041235 | 0.007 |
| FGF20 | C8orf49 | hsa-mir-100 | 0.042686 | 0.007 |
| GUCY1A2 | MEG3 | hsa-mir-508 | 0.02387 | 0.007 |
| HMCN2 | LINC00470 | hsa-mir-424 | 0.029757 | 0.007 |
| HRH3 | KIAA0087 | hsa-mir-145 | 0.045807 | 0.007 |
| KCTD12 | MAGI2-AS3 | hsa-mir-210 | 0.071194 | 0.007 |
| KIAA0087 | PPP1R12B | hsa-mir-141 | 0.052315 | 0.007 |
| LDB2 | CMAHP | hsa-mir-424 | 0.034126 | 0.007 |
| LHX3 | NAV2-AS2 | hsa-mir-96 | 0.027782 | 0.007 |
| LINC00051 | KRTAP4-9 | hsa-mir-205 | 0.03195 | 0.007 |
| LINC00355 | PDLIM3 | hsa-mir-195 | 0.041058 | 0.007 |
| LINC00355 | TNS1 | hsa-mir-195 | 0.034146 | 0.007 |
| LINC00355 | DGKB | hsa-mir-141 | 0.040817 | 0.007 |
| LINC00470 | KCNS2 | hsa-mir-424 | 0.055745 | 0.007 |
| LINC00483 | MEF2C | hsa-mir-215 | 0.031933 | 0.007 |
| LINC00483 | HAND2 | hsa-mir-215 | 0.027991 | 0.007 |
| LINC00483 | EDNRA | hsa-mir-215 | 0.044685 | 0.007 |
| MAGI2-AS3 | NXPH3 | hsa-mir-216b | 0.033522 | 0.007 |
| MAGI2-AS3 | WT1 | hsa-mir-429 | 0.063939 | 0.007 |
| MAGI2-AS3 | KLHL4 | hsa-mir-210 | 0.049611 | 0.007 |
| MAGI2-AS3 | MYCT1 | hsa-mir-210 | 0.080833 | 0.007 |
| MASP1 | LINC00491 | hsa-mir-145 | 0.030522 | 0.007 |
| MRVI1 | LINC00470 | hsa-mir-143 | 0.030217 | 0.007 |
| MSRB3 | AGAP11 | hsa-mir-200a | 0.043314 | 0.007 |
| MSRB3 | AGAP11 | hsa-mir-424 | 0.048617 | 0.007 |
| MUC2 | FRMD6 | hsa-mir-106a | 0.02315 | 0.007 |
| MUC2 | E2F2 | hsa-mir-182 | 0.042547 | 0.007 |
| NEUROD1 | MIR210HG | hsa-mir-424 | 0.035021 | 0.007 |
| NR3C1 | LINC00470 | hsa-mir-508 | 0.02695 | 0.007 |
| PGM5 | LINC00314 | hsa-mir-143 | 0.039862 | 0.007 |
| POU6F2 | NOVA1-AS1 | hsa-mir-489 | 0.026286 | 0.007 |
| RMST | SCGN | hsa-mir-211 | 0.047859 | 0.007 |
| SELP | LINC00314 | hsa-mir-143 | 0.03918 | 0.007 |
| SLITRK3 | LINC00211 | hsa-mir-301b | 0.037295 | 0.007 |
| SMIM10 | C8orf49 | hsa-mir-424 | 0.06673 | 0.007 |
| SYNPO2 | LINC00473 | hsa-mir-195 | 0.035705 | 0.007 |
| TCEAL7 | LINC00470 | hsa-mir-143 | 0.034957 | 0.007 |
| TSHZ3 | AC135178.1 | hsa-mir-122 | 0.065895 | 0.007 |
| ZNF366 | MEG3 | hsa-mir-205 | 0.037256 | 0.007 |
| ABCG2 | C20orf166-AS1 | hsa-mir-183 | 0.035323 | 0.006 |
| ADAMTS9-AS2 | TNS1 | hsa-mir-205 | 0.030098 | 0.006 |
| ADAMTS9-AS2 | AQP1 | hsa-mir-96 | 0.025132 | 0.006 |
| ADAMTS9-AS2 | YPEL4 | hsa-mir-96 | 0.051496 | 0.006 |
| ADRB3 | NOVA1-AS1 | hsa-mir-489 | 0.027709 | 0.006 |
| AGAP11 | FAM83C | hsa-mir-216b | 0.039487 | 0.006 |
| AGAP11 | CHRNA4 | hsa-mir-204 | 0.022667 | 0.006 |
| AKT3 | AC020907.1 | hsa-mir-424 | 0.036998 | 0.006 |
| AL713998.1 | GPRASP1 | hsa-mir-141 | 0.042445 | 0.006 |
| ARHGEF15 | NKX2-1-AS1 | hsa-mir-211 | 0.03678 | 0.006 |
| ATXN8OS | LHX3 | hsa-mir-210 | 0.027222 | 0.006 |
| C8orf49 | IRS4 | hsa-mir-143 | 0.062213 | 0.006 |
| C8orf49 | XIRP2 | hsa-mir-195 | 0.044885 | 0.006 |
| CLMP | DIO3OS | hsa-mir-143 | 0.050316 | 0.006 |
| CLMP | LINC00470 | hsa-mir-424 | 0.033422 | 0.006 |
| CMAHP | SEZ6L | hsa-mir-141 | 0.037078 | 0.006 |
| CNRIP1 | CLRN1-AS1 | hsa-mir-489 | 0.02681 | 0.006 |
| CNTN4 | MEG3 | hsa-mir-508 | 0.026177 | 0.006 |
| CPED1 | LINC00211 | hsa-mir-204 | 0.028038 | 0.006 |
| CSDC2 | DIO3OS | hsa-mir-143 | 0.050617 | 0.006 |
| FAM9B | AGAP11 | hsa-mir-141 | 0.030181 | 0.006 |
| GLI2 | WT1-AS | hsa-mir-216b | 0.034337 | 0.006 |
| HCG23 | NKX2-1 | hsa-mir-106a | 0.041457 | 0.006 |
| JAM3 | C8orf49 | hsa-mir-424 | 0.068453 | 0.006 |
| KIAA1462 | C2orf48 | hsa-mir-106a | 0.02887 | 0.006 |
| KIAA1462 | C8orf49 | hsa-mir-143 | 0.040567 | 0.006 |
| LEPR | CMAHP | hsa-mir-424 | 0.038462 | 0.006 |
| LIFR | FAM41C | hsa-mir-200a | 0.042729 | 0.006 |
| LINC00211 | KLHL1 | hsa-mir-216b | 0.037405 | 0.006 |
| LINC00314 | OR5C1 | hsa-mir-211 | 0.039438 | 0.006 |
| LINC00337 | C1QTNF7 | hsa-mir-383 | 0.104136 | 0.006 |
| LINC00458 | SALL3 | hsa-mir-143 | 0.039711 | 0.006 |
| LINC00470 | EHD2 | hsa-mir-424 | 0.150728 | 0.006 |
| LINC00470 | PIF1 | hsa-mir-216b | 0.028728 | 0.006 |
| LINC00483 | VWC2 | hsa-mir-215 | 0.028424 | 0.006 |
| LINC00523 | OPCML | hsa-mir-200a | 0.055246 | 0.006 |
| MAGI2-AS3 | SPARCL1 | hsa-mir-106a | 0.037961 | 0.006 |
| MAGI2-AS3 | KCNA1 | hsa-mir-424 | 0.047492 | 0.006 |
| MEG3 | MYOCD | hsa-mir-301b | 0.044266 | 0.006 |
| MEG3 | SYNPO2 | hsa-mir-205 | 0.035345 | 0.006 |
| MIR7-3HG | MLNR | hsa-mir-211 | 0.030789 | 0.006 |
| MRVI1 | C8orf49 | hsa-mir-143 | 0.042001 | 0.006 |
| NAV2-AS2 | LHX3 | hsa-mir-96 | 0.026916 | 0.006 |
| PDE8B | WT1-AS | hsa-mir-145 | 0.142579 | 0.006 |
| PPP1R12B | LINC00470 | hsa-mir-143 | 0.032891 | 0.006 |
| PPP1R12B | MUC2 | hsa-mir-140 | 0.036475 | 0.006 |
| PURG | C20orf166-AS1 | hsa-mir-489 | 0.034007 | 0.006 |
| RHOJ | LINC00237 | hsa-mir-143 | 0.030298 | 0.006 |
| SLITRK3 | MEG3 | hsa-mir-211 | 0.038916 | 0.006 |
| SORBS1 | C2orf48 | hsa-mir-106a | 0.023662 | 0.006 |
| TAL1 | MEG3 | hsa-mir-508 | 0.024047 | 0.006 |
| TCEAL6 | DLX6-AS1 | hsa-mir-145 | 0.039254 | 0.006 |
| TCEAL7 | KIAA0087 | hsa-mir-141 | 0.036068 | 0.006 |
| TFAP2B | CLRN1-AS1 | hsa-mir-211 | 0.025208 | 0.006 |
| TSPAN2 | LINC00314 | hsa-mir-143 | 0.039962 | 0.006 |
| UGT1A10 | RMST | hsa-mir-211 | 0.039153 | 0.006 |
| WT1-AS | MASP1 | hsa-mir-363 | 0.024869 | 0.006 |
| WT1-AS | LMOD1 | hsa-mir-106a | 0.028438 | 0.006 |
| WT1-AS | ANTXR2 | hsa-mir-145 | 0.156601 | 0.006 |
| ZCCHC24 | C2orf48 | hsa-mir-424 | 0.025262 | 0.006 |
| ZNF366 | KIAA0087 | hsa-mir-211 | 0.041432 | 0.006 |
| ADARB2-AS1 | FIBCD1 | hsa-mir-205 | 0.025826 | 0.005 |
| ADH1B | LINC00314 | hsa-mir-143 | 0.034758 | 0.005 |
| AGAP11 | FAM9B | hsa-mir-141 | 0.052321 | 0.005 |
| AGAP11 | C3orf70 | hsa-mir-424 | 0.059593 | 0.005 |
| ALPP | C8orf49 | hsa-mir-301b | 0.024954 | 0.005 |
| ANTXR2 | AGAP11 | hsa-mir-424 | 0.05111 | 0.005 |
| BX255923.1 | ABCG2 | hsa-mir-211 | 0.028502 | 0.005 |
| C8orf49 | CAMK2A | hsa-mir-143 | 0.102459 | 0.005 |
| C8orf49 | FGF20 | hsa-mir-100 | 0.036021 | 0.005 |
| C8orf49 | ASPA | hsa-mir-143 | 0.052777 | 0.005 |
| CCL14 | LINC00491 | hsa-mir-145 | 0.027045 | 0.005 |
| CDON | C8orf49 | hsa-mir-429 | 0.037638 | 0.005 |
| CLMP | LINC00491 | hsa-mir-145 | 0.028528 | 0.005 |
| CLRN1-AS1 | CNRIP1 | hsa-mir-489 | 0.027688 | 0.005 |
| DIO3OS | CPED1 | hsa-mir-143 | 0.059775 | 0.005 |
| FRMD6-AS2 | CFHR4 | hsa-mir-96 | 0.025427 | 0.005 |
| FRMD6-AS2 | TMPRSS15 | hsa-mir-211 | 0.061381 | 0.005 |
| GAD2 | LINC00355 | hsa-mir-424 | 0.032139 | 0.005 |
| GLI2 | C15orf54 | hsa-mir-424 | 0.027596 | 0.005 |
| GLT1D1 | WT1-AS | hsa-mir-195 | 0.051196 | 0.005 |
| GRK5 | DIO3OS | hsa-mir-211 | 0.021738 | 0.005 |
| GSTM5 | LINC00470 | hsa-mir-424 | 0.034204 | 0.005 |
| HSD17B13 | KIAA0087 | hsa-mir-195 | 0.040419 | 0.005 |
| KLF17 | BX255923.1 | hsa-mir-204 | 0.047206 | 0.005 |
| LINC00211 | SPINK8 | hsa-mir-508 | 0.033877 | 0.005 |
| LINC00393 | KDR | hsa-mir-106a | 0.032249 | 0.005 |
| LINC00458 | HRH3 | hsa-mir-205 | 0.024913 | 0.005 |
| LINC00483 | NEUROD1 | hsa-mir-216b | 0.041021 | 0.005 |
| LINC00523 | KLHL1 | hsa-mir-122 | 0.081281 | 0.005 |
| MASP1 | LINC00470 | hsa-mir-143 | 0.033691 | 0.005 |
| MEG3 | GUCY1A2 | hsa-mir-205 | 0.042671 | 0.005 |
| MEG3 | GPR22 | hsa-mir-205 | 0.043264 | 0.005 |
| MRGPRF | LINC00237 | hsa-mir-143 | 0.029383 | 0.005 |
| MRVI1 | WT1-AS | hsa-mir-106a | 0.052842 | 0.005 |
| MUC2 | TCEAL6 | hsa-mir-140 | 0.056814 | 0.005 |
| MUC2 | WFDC5 | hsa-mir-195 | 0.036506 | 0.005 |
| MYF5 | LINC00314 | hsa-mir-106a | 0.032605 | 0.005 |
| MYOCD | LINC00314 | hsa-mir-143 | 0.045167 | 0.005 |
| NAV2-AS2 | GUCY1A2 | hsa-mir-182 | 0.036362 | 0.005 |
| NAV2-AS2 | DDR2 | hsa-mir-182 | 0.030811 | 0.005 |
| NKX2-1-AS1 | VSX2 | hsa-mir-96 | 0.025049 | 0.005 |
| PLN | AGAP11 | hsa-mir-424 | 0.045723 | 0.005 |
| PRLHR | LINC00314 | hsa-mir-143 | 0.036183 | 0.005 |
| PTGER3 | LINC00314 | hsa-mir-143 | 0.045126 | 0.005 |
| PURG | LINC00470 | hsa-mir-424 | 0.032087 | 0.005 |
| RASGRP2 | LINC00470 | hsa-mir-424 | 0.036831 | 0.005 |
| RMRP | KRTAP4-9 | hsa-mir-122 | 0.070812 | 0.005 |
| SPINK8 | LINC00211 | hsa-mir-508 | 0.030039 | 0.005 |
| SYNPO2 | NAV2-AS2 | hsa-mir-182 | 0.037385 | 0.005 |
| SYNPO2 | CMAHP | hsa-mir-424 | 0.04069 | 0.005 |
| TCF21 | LINC00470 | hsa-mir-424 | 0.034521 | 0.005 |
| TCF23 | LINC00314 | hsa-mir-143 | 0.036034 | 0.005 |
| TMEM200B | LINC00314 | hsa-mir-143 | 0.040616 | 0.005 |
| VSX2 | HCG23 | hsa-mir-145 | 0.050733 | 0.005 |
| WT1-AS | KLHDC8A | hsa-mir-383 | 0.034001 | 0.005 |
| WT1-AS | PGM5 | hsa-mir-106a | 0.035418 | 0.005 |
| ZFPM2 | MEG3 | hsa-mir-508 | 0.029292 | 0.005 |
| ADAMTS9-AS2 | CXCL12 | hsa-mir-205 | 0.031881 | 0.004 |
| AGAP11 | NEUROD1 | hsa-mir-204 | 0.042439 | 0.004 |
| AQP1 | LINC00237 | hsa-mir-143 | 0.030262 | 0.004 |
| ASPN | WT1-AS | hsa-mir-363 | 0.036115 | 0.004 |
| ATXN8OS | VRTN | hsa-mir-211 | 0.045724 | 0.004 |
| C10orf126 | KRTAP4-9 | hsa-mir-363 | 0.028777 | 0.004 |
| C10orf126 | AMBN | hsa-mir-140 | 0.06729 | 0.004 |
| C20orf166-AS1 | PURG | hsa-mir-489 | 0.040827 | 0.004 |
| C6orf99 | GLI2 | hsa-mir-140 | 0.049687 | 0.004 |
| CLRN1-AS1 | TRPM1 | hsa-mir-140 | 0.024 | 0.004 |
| CPED1 | FAM41C | hsa-mir-200a | 0.047676 | 0.004 |
| CRCT1 | FRMD6-AS2 | hsa-mir-211 | 0.045364 | 0.004 |
| DIO3OS | MEF2C | hsa-mir-143 | 0.085556 | 0.004 |
| DIO3OS | SHE | hsa-mir-143 | 0.07161 | 0.004 |
| DIXDC1 | C20orf166-AS1 | hsa-mir-183 | 0.034881 | 0.004 |
| DPYSL5 | RMST | hsa-mir-205 | 0.029572 | 0.004 |
| ECM2 | LINC00470 | hsa-mir-424 | 0.034675 | 0.004 |
| EMCN | LINC00491 | hsa-mir-145 | 0.033372 | 0.004 |
| GPR52 | LINC00211 | hsa-mir-183 | 0.027401 | 0.004 |
| GRM7 | LINC00470 | hsa-mir-424 | 0.040336 | 0.004 |
| GUCY1A2 | MEG3 | hsa-mir-205 | 0.039473 | 0.004 |
| HAND2 | LINC00470 | hsa-mir-424 | 0.035265 | 0.004 |
| ITGA8 | WT1-AS | hsa-mir-145 | 0.147132 | 0.004 |
| JAZF1 | MEG3 | hsa-mir-301b | 0.033784 | 0.004 |
| KDR | LINC00393 | hsa-mir-106a | 0.036991 | 0.004 |
| KIAA0087 | ABCG2 | hsa-mir-211 | 0.025372 | 0.004 |
| KIAA0087 | BSPRY | hsa-mir-182 | 0.082127 | 0.004 |
| KIAA0087 | ZNF366 | hsa-mir-211 | 0.043785 | 0.004 |
| KIAA1462 | WT1-AS | hsa-mir-145 | 0.140861 | 0.004 |
| KLHL1 | LINC00523 | hsa-mir-122 | 0.086236 | 0.004 |
| KLHL4 | LINC00314 | hsa-mir-143 | 0.042548 | 0.004 |
| KRTAP11-1 | LINC00314 | hsa-mir-204 | 0.033042 | 0.004 |
| KRTAP3-2 | RMRP | hsa-mir-122 | 0.062159 | 0.004 |
| LINC00051 | BRS3 | hsa-mir-205 | 0.034732 | 0.004 |
| LINC00211 | TPTE | hsa-mir-301b | 0.032108 | 0.004 |
| LINC00337 | LHFP | hsa-mir-383 | 0.046928 | 0.004 |
| LINC00355 | BTBD17 | hsa-mir-122 | 0.048106 | 0.004 |
| LMOD1 | LINC00237 | hsa-mir-143 | 0.038066 | 0.004 |
| MAGI2-AS3 | CLMP | hsa-mir-210 | 0.061484 | 0.004 |
| MEG3 | CNTN4 | hsa-mir-508 | 0.030632 | 0.004 |
| MEG3 | PDYN | hsa-mir-424 | 0.051364 | 0.004 |
| MITF | CLRN1-AS1 | hsa-mir-140 | 0.045525 | 0.004 |
| MRGPRF | FAM41C | hsa-mir-200a | 0.051086 | 0.004 |
| MRVI1 | MEG3 | hsa-mir-205 | 0.037028 | 0.004 |
| MYOCD | MUC2 | hsa-mir-140 | 0.036166 | 0.004 |
| NKX2-1 | HCG23 | hsa-mir-106a | 0.046847 | 0.004 |
| NKX2-1-AS1 | HRH3 | hsa-mir-145 | 0.027726 | 0.004 |
| PIF1 | LINC00470 | hsa-mir-216b | 0.031375 | 0.004 |
| RSPO1 | DIO3OS | hsa-mir-143 | 0.05706 | 0.004 |
| S100A7A | KIAA0087 | hsa-mir-424 | 0.02815 | 0.004 |
| SYNPO2 | WT1-AS | hsa-mir-106a | 0.051444 | 0.004 |
| TCEAL6 | MUC2 | hsa-mir-140 | 0.037621 | 0.004 |
| TCEAL7 | DIO3OS | hsa-mir-143 | 0.055584 | 0.004 |
| TCF23 | C8orf49 | hsa-mir-143 | 0.049673 | 0.004 |
| TGFB1I1 | LINC00237 | hsa-mir-143 | 0.033953 | 0.004 |
| TNS1 | LINC00470 | hsa-mir-143 | 0.039801 | 0.004 |
| TSHZ3 | AC092422.1 | hsa-mir-424 | 0.042505 | 0.004 |
| WT1 | C20orf166-AS1 | hsa-mir-106a | 0.030809 | 0.004 |
| WT1-AS | ABCG2 | hsa-mir-106a | 0.044279 | 0.004 |
| WT1-AS | TMEM196 | hsa-mir-145 | 0.050277 | 0.004 |
| AC135178.1 | TSHZ3 | hsa-mir-122 | 0.042148 | 0.003 |
| ADAMTS9-AS2 | ZNF540 | hsa-mir-96 | 0.033979 | 0.003 |
| AGAP11 | B3GNT4 | hsa-mir-211 | 0.025909 | 0.003 |
| AL713998.1 | IRS4 | hsa-mir-141 | 0.054332 | 0.003 |
| BPIFA1 | NAV2-AS2 | hsa-mir-204 | 0.048525 | 0.003 |
| C15orf54 | MAGEC2 | hsa-mir-182 | 0.042017 | 0.003 |
| C8orf49 | NEUROD1 | hsa-mir-106a | 0.037912 | 0.003 |
| CAMK2A | C8orf49 | hsa-mir-143 | 0.048493 | 0.003 |
| CLRN1-AS1 | KRTAP4-6 | hsa-mir-195 | 0.039202 | 0.003 |
| CPED1 | FAM41C | hsa-mir-145 | 0.053861 | 0.003 |
| CSDC2 | MEG3 | hsa-mir-301b | 0.042926 | 0.003 |
| DGKB | LINC00473 | hsa-mir-195 | 0.035926 | 0.003 |
| DIO3OS | DGKB | hsa-mir-143 | 0.126085 | 0.003 |
| DPYSL5 | LINC00355 | hsa-mir-424 | 0.03479 | 0.003 |
| DSCR4-IT1 | KRT74 | hsa-mir-211 | 0.050737 | 0.003 |
| GIMAP1 | C20orf166-AS1 | hsa-mir-489 | 0.0378 | 0.003 |
| GPR83 | C10orf126 | hsa-mir-140 | 0.033529 | 0.003 |
| IRS4 | C8orf49 | hsa-mir-143 | 0.044034 | 0.003 |
| LEPR | LINC00314 | hsa-mir-143 | 0.0394 | 0.003 |
| LHFP | LINC00337 | hsa-mir-383 | 0.031083 | 0.003 |
| LHX3 | ATXN8OS | hsa-mir-210 | 0.034571 | 0.003 |
| LHX3 | C8orf49 | hsa-mir-106a | 0.052303 | 0.003 |
| LHX3 | LINC00458 | hsa-mir-200a | 0.030835 | 0.003 |
| LINC00051 | TPTE | hsa-mir-363 | 0.031022 | 0.003 |
| LINC00052 | HOXC13 | hsa-mir-425 | 0.02649 | 0.003 |
| LINC00113 | GLI2 | hsa-mir-145 | 0.139797 | 0.003 |
| LINC00488 | TNS1 | hsa-mir-205 | 0.032027 | 0.003 |
| LMOD1 | WT1-AS | hsa-mir-363 | 0.038668 | 0.003 |
| LPP | LINC00355 | hsa-mir-195 | 0.040953 | 0.003 |
| MAGI2-AS3 | DDR2 | hsa-mir-210 | 0.062816 | 0.003 |
| MAGI2-AS3 | MEF2C | hsa-mir-210 | 0.078363 | 0.003 |
| MASP1 | LINC00314 | hsa-mir-143 | 0.04906 | 0.003 |
| MEF2C | AGAP11 | hsa-mir-200a | 0.055567 | 0.003 |
| MEG3 | DDR2 | hsa-mir-508 | 0.028912 | 0.003 |
| MEG3 | ZNF366 | hsa-mir-205 | 0.028749 | 0.003 |
| MEG3 | GUCY1A2 | hsa-mir-508 | 0.030065 | 0.003 |
| MEG3 | C1QTNF7 | hsa-mir-508 | 0.041144 | 0.003 |
| MEG3 | CLMP | hsa-mir-508 | 0.034981 | 0.003 |
| MRVI1 | LINC00355 | hsa-mir-195 | 0.047088 | 0.003 |
| MSRB3 | LINC00314 | hsa-mir-143 | 0.043766 | 0.003 |
| MYH11 | DLX6-AS1 | hsa-mir-211 | 0.032676 | 0.003 |
| MYOCD | LINC00211 | hsa-mir-204 | 0.026261 | 0.003 |
| NDN | LINC00491 | hsa-mir-145 | 0.029722 | 0.003 |
| NEUROD1 | AGAP11 | hsa-mir-216b | 0.072501 | 0.003 |
| NXPH3 | MAGI2-AS3 | hsa-mir-216b | 0.042496 | 0.003 |
| NXPH3 | LINC00458 | hsa-mir-204 | 0.027735 | 0.003 |
| OLFML1 | MEG3 | hsa-mir-508 | 0.029039 | 0.003 |
| PLN | WT1-AS | hsa-mir-363 | 0.044581 | 0.003 |
| PLN | LINC00470 | hsa-mir-143 | 0.042486 | 0.003 |
| PPP1R12B | WT1-AS | hsa-mir-106a | 0.058803 | 0.003 |
| PPP1R12B | DLX6-AS1 | hsa-mir-145 | 0.043814 | 0.003 |
| PPP1R12B | FAM41C | hsa-mir-145 | 0.049973 | 0.003 |
| PRKG1 | NAV2-AS2 | hsa-mir-182 | 0.040555 | 0.003 |
| PTPRB | FAM41C | hsa-mir-145 | 0.054774 | 0.003 |
| RMST | RSPO3 | hsa-mir-301b | 0.033777 | 0.003 |
| SPHKAP | MAGI2-AS3 | hsa-mir-424 | 0.067986 | 0.003 |
| SYNPO2 | CLRN1-AS1 | hsa-mir-489 | 0.030711 | 0.003 |
| TCEAL7 | CMAHP | hsa-mir-424 | 0.04039 | 0.003 |
| TIMP3 | ADAMTS9-AS2 | hsa-mir-200a | 0.041574 | 0.003 |
| TMPRSS15 | FRMD6-AS2 | hsa-mir-211 | 0.043182 | 0.003 |
| TNS1 | LINC00488 | hsa-mir-205 | 0.024044 | 0.003 |
| TPTE | LINC00211 | hsa-mir-301b | 0.034104 | 0.003 |
| VSTM4 | CMAHP | hsa-mir-195 | 0.051716 | 0.003 |
| ABCG2 | WT1-AS | hsa-mir-106a | 0.053023 | 0.002 |
| AC020907.1 | TNS1 | hsa-mir-424 | 0.068649 | 0.002 |
| AC135178.1 | CNRIP1 | hsa-mir-122 | 0.040749 | 0.002 |
| ADAMTS9-AS2 | PAMR1 | hsa-mir-96 | 0.066228 | 0.002 |
| ADARB2-AS1 | SORCS3 | hsa-mir-195 | 0.03246 | 0.002 |
| ADARB2-AS1 | FAM9C | hsa-mir-205 | 0.035136 | 0.002 |
| AGAP11 | PPP1R12B | hsa-mir-424 | 0.06202 | 0.002 |
| AGAP11 | KRT74 | hsa-mir-205 | 0.077325 | 0.002 |
| BRS3 | LINC00051 | hsa-mir-140 | 0.05942 | 0.002 |
| BSPRY | KIAA0087 | hsa-mir-182 | 0.035538 | 0.002 |
| BX255923.1 | MYCT1 | hsa-mir-211 | 0.035194 | 0.002 |
| C1QTNF7 | C20orf166-AS1 | hsa-mir-489 | 0.035959 | 0.002 |
| C1QTNF7 | MEG3 | hsa-mir-508 | 0.032864 | 0.002 |
| C20orf166-AS1 | GIMAP1 | hsa-mir-489 | 0.034745 | 0.002 |
| C8orf49 | CLDN19 | hsa-mir-429 | 0.059524 | 0.002 |
| CDHR5 | MAGI2-AS3 | hsa-mir-195 | 0.037017 | 0.002 |
| CLRN1-AS1 | SYNPO2 | hsa-mir-489 | 0.037479 | 0.002 |
| CLRN1-AS1 | OLIG3 | hsa-mir-424 | 0.032095 | 0.002 |
| CNGA2 | ATXN8OS | hsa-mir-211 | 0.071471 | 0.002 |
| CPED1 | BX255923.1 | hsa-mir-211 | 0.065346 | 0.002 |
| CPED1 | MEG3 | hsa-mir-508 | 0.03543 | 0.002 |
| DDR2 | MEG3 | hsa-mir-508 | 0.031466 | 0.002 |
| DIO3OS | OLFML2A | hsa-mir-143 | 0.073702 | 0.002 |
| DIO3OS | SLC8A1 | hsa-mir-383 | 0.052195 | 0.002 |
| E2F2 | AC107959.1 | hsa-mir-301b | 0.032956 | 0.002 |
| EDNRA | LINC00470 | hsa-mir-424 | 0.039657 | 0.002 |
| EMILIN1 | AGAP11 | hsa-mir-200a | 0.051475 | 0.002 |
| EN1 | JAZF1-AS1 | hsa-mir-205 | 0.037007 | 0.002 |
| FAM41C | LIFR | hsa-mir-200a | 0.049016 | 0.002 |
| FBXL7 | LINC00470 | hsa-mir-424 | 0.042032 | 0.002 |
| FBXO40 | LINC00314 | hsa-mir-211 | 0.062331 | 0.002 |
| FLRT2 | LINC00470 | hsa-mir-424 | 0.044987 | 0.002 |
| FRMD6-AS2 | CRCT1 | hsa-mir-211 | 0.05221 | 0.002 |
| GUCA2A | AGAP11 | hsa-mir-195 | 0.030934 | 0.002 |
| HSPB7 | LINC00355 | hsa-mir-195 | 0.053499 | 0.002 |
| IFFO1 | LINC00470 | hsa-mir-424 | 0.044657 | 0.002 |
| IRS4 | AL713998.1 | hsa-mir-141 | 0.035722 | 0.002 |
| KCNQ1DN | MAGEC2 | hsa-mir-195 | 0.106576 | 0.002 |
| KIAA1644 | C8orf49 | hsa-mir-143 | 0.044418 | 0.002 |
| KLHL38 | LINC00473 | hsa-mir-195 | 0.040225 | 0.002 |
| KRT85 | LINC00314 | hsa-mir-204 | 0.039233 | 0.002 |
| KRTAP4-9 | RMRP | hsa-mir-122 | 0.079413 | 0.002 |
| LHX3 | C8orf49 | hsa-mir-429 | 0.057503 | 0.002 |
| LINC00051 | BRS3 | hsa-mir-140 | 0.076425 | 0.002 |
| LINC00211 | XIRP2 | hsa-mir-216b | 0.046857 | 0.002 |
| LINC00314 | LEPR | hsa-mir-143 | 0.065931 | 0.002 |
| LINC00314 | KRT85 | hsa-mir-204 | 0.056146 | 0.002 |
| LINC00355 | HSPB7 | hsa-mir-195 | 0.070903 | 0.002 |
| LINC00355 | PCSK2 | hsa-mir-424 | 0.035006 | 0.002 |
| LINC00458 | EMX1 | hsa-mir-143 | 0.034607 | 0.002 |
| LINC00458 | PRR9 | hsa-mir-204 | 0.072195 | 0.002 |
| LINC00458 | KRT74 | hsa-mir-204 | 0.05735 | 0.002 |
| LINC00470 | LRRN4CL | hsa-mir-424 | 0.114949 | 0.002 |
| LINC00470 | MYF5 | hsa-mir-508 | 0.031335 | 0.002 |
| LINC00470 | PAX1 | hsa-mir-508 | 0.030279 | 0.002 |
| LINC00473 | PLN | hsa-mir-195 | 0.111665 | 0.002 |
| LMOD1 | LINC00314 | hsa-mir-143 | 0.054689 | 0.002 |
| MAGEC2 | LINC00052 | hsa-mir-363 | 0.036824 | 0.002 |
| MAGI2-AS3 | SPHKAP | hsa-mir-424 | 0.067251 | 0.002 |
| MAGI2-AS3 | TSHZ3 | hsa-mir-106a | 0.045225 | 0.002 |
| MAGI2-AS3 | ADTRP | hsa-mir-143 | 0.048513 | 0.002 |
| MAGI2-AS3 | CDHR5 | hsa-mir-195 | 0.03291 | 0.002 |
| MAGI2-AS3 | OLFML1 | hsa-mir-210 | 0.064892 | 0.002 |
| MASP1 | C8orf49 | hsa-mir-143 | 0.049977 | 0.002 |
| MEG3 | EMILIN1 | hsa-mir-508 | 0.041828 | 0.002 |
| MEG3 | ASPA | hsa-mir-205 | 0.038277 | 0.002 |
| MEG3 | CSDC2 | hsa-mir-301b | 0.048425 | 0.002 |
| MEG3 | MRVI1 | hsa-mir-205 | 0.041285 | 0.002 |
| MEG3 | OLFML1 | hsa-mir-508 | 0.034498 | 0.002 |
| MRVI1 | AGAP11 | hsa-mir-424 | 0.057697 | 0.002 |
| MYF5 | LINC00470 | hsa-mir-508 | 0.03167 | 0.002 |
| MYOCD | LINC00355 | hsa-mir-195 | 0.052139 | 0.002 |
| OGN | LINC00237 | hsa-mir-143 | 0.033234 | 0.002 |
| OLFML2A | DIO3OS | hsa-mir-143 | 0.057016 | 0.002 |
| PAMR1 | ADAMTS9-AS2 | hsa-mir-96 | 0.052148 | 0.002 |
| PBOV1 | LINC00211 | hsa-mir-183 | 0.033762 | 0.002 |
| PBX1 | RERG-IT1 | hsa-mir-182 | 0.044577 | 0.002 |
| PCSK2 | LINC00355 | hsa-mir-424 | 0.040453 | 0.002 |
| PDLIM3 | LINC00355 | hsa-mir-195 | 0.046898 | 0.002 |
| PPP1R12B | KIAA0087 | hsa-mir-141 | 0.031361 | 0.002 |
| PPP1R12B | LINC00314 | hsa-mir-143 | 0.046845 | 0.002 |
| PTGFR | C8orf49 | hsa-mir-143 | 0.053104 | 0.002 |
| SHE | DIO3OS | hsa-mir-143 | 0.058683 | 0.002 |
| SLC8A1 | DIO3OS | hsa-mir-383 | 0.042421 | 0.002 |
| SLITRK3 | NOVA1-AS1 | hsa-mir-489 | 0.032026 | 0.002 |
| SOX21-AS1 | SOX21 | hsa-mir-301b | 0.061784 | 0.002 |
| SYNPO2 | RMST | hsa-mir-195 | 0.032782 | 0.002 |
| SYNPO2 | C8orf49 | hsa-mir-143 | 0.048241 | 0.002 |
| TMEM200B | WT1-AS | hsa-mir-363 | 0.044201 | 0.002 |
| TNS1 | WT1-AS | hsa-mir-106a | 0.063006 | 0.002 |
| TSHZ3 | MAGI2-AS3 | hsa-mir-106a | 0.035642 | 0.002 |
| TSHZ3 | AGAP11 | hsa-mir-200a | 0.045664 | 0.002 |
| VRTN | ATXN8OS | hsa-mir-211 | 0.067704 | 0.002 |
| WFDC5 | MUC2 | hsa-mir-195 | 0.044725 | 0.002 |
| WT1-AS | LEPR | hsa-mir-145 | 0.076233 | 0.002 |
| WT1-AS | SYNPO2 | hsa-mir-363 | 0.037026 | 0.002 |
| WT1-AS | PTPRB | hsa-mir-106a | 0.035706 | 0.002 |
| WT1-AS | GLT1D1 | hsa-mir-195 | 0.055474 | 0.002 |
| WT1-AS | PDE8B | hsa-mir-145 | 0.100933 | 0.002 |
| WT1-AS | TCEAL7 | hsa-mir-106a | 0.044698 | 0.002 |
| ZNF366 | C8orf49 | hsa-mir-143 | 0.042344 | 0.002 |
| ABCC9 | MAGI2-AS3 | hsa-mir-106a | 0.049477 | 0.001 |
| ADARB2-AS1 | SPRR2G | hsa-mir-205 | 0.069292 | 0.001 |
| ADARB2-AS1 | HOXD12 | hsa-mir-205 | 0.046534 | 0.001 |
| ADH1B | LINC00491 | hsa-mir-145 | 0.035204 | 0.001 |
| AGAP11 | TCF23 | hsa-mir-424 | 0.061735 | 0.001 |
| AGAP11 | MEF2C | hsa-mir-200a | 0.057111 | 0.001 |
| AGAP11 | NEUROD1 | hsa-mir-216b | 0.069425 | 0.001 |
| AKAP2 | BX255923.1 | hsa-mir-211 | 0.076338 | 0.001 |
| ALPP | KIAA0087 | hsa-mir-96 | 0.031017 | 0.001 |
| AMBN | C10orf126 | hsa-mir-140 | 0.036813 | 0.001 |
| ATXN8OS | NPFFR2 | hsa-mir-204 | 0.046504 | 0.001 |
| ATXN8OS | LHX3 | hsa-mir-211 | 0.070649 | 0.001 |
| ATXN8OS | CNGA2 | hsa-mir-211 | 0.074843 | 0.001 |
| ATXN8OS | CNGA2 | hsa-mir-210 | 0.055234 | 0.001 |
| AXL | LINC00470 | hsa-mir-424 | 0.046899 | 0.001 |
| BRS3 | LINC00314 | hsa-mir-204 | 0.057791 | 0.001 |
| BRS3 | LINC00523 | hsa-mir-508 | 0.054972 | 0.001 |
| BRS3 | RERG-IT1 | hsa-mir-182 | 0.071972 | 0.001 |
| BTBD17 | LINC00355 | hsa-mir-122 | 0.071154 | 0.001 |
| BTBD17 | LINC00458 | hsa-mir-200a | 0.040041 | 0.001 |
| BX255923.1 | CPED1 | hsa-mir-211 | 0.055651 | 0.001 |
| BX255923.1 | AKAP2 | hsa-mir-211 | 0.045704 | 0.001 |
| BX255923.1 | KLF17 | hsa-mir-204 | 0.048143 | 0.001 |
| BX255923.1 | FBXO40 | hsa-mir-204 | 0.0621 | 0.001 |
| C10orf126 | TMPRSS11A | hsa-mir-141 | 0.059077 | 0.001 |
| C10orf126 | CST5 | hsa-mir-140 | 0.061303 | 0.001 |
| C1orf141 | CLRN1-AS1 | hsa-mir-211 | 0.053841 | 0.001 |
| C1QTNF7 | LINC00337 | hsa-mir-383 | 0.032604 | 0.001 |
| C3orf70 | DIO3OS | hsa-mir-143 | 0.089981 | 0.001 |
| C8orf49 | WFDC5 | hsa-mir-301b | 0.055349 | 0.001 |
| C8orf49 | LHX3 | hsa-mir-429 | 0.046834 | 0.001 |
| C8orf49 | LHX3 | hsa-mir-106a | 0.062551 | 0.001 |
| C8orf49 | KLF17 | hsa-mir-122 | 0.040181 | 0.001 |
| C8orf49 | FBXO40 | hsa-mir-122 | 0.049727 | 0.001 |
| C8orf49 | CNGA2 | hsa-mir-106a | 0.054506 | 0.001 |
| C8orf49 | VRTN | hsa-mir-106a | 0.053855 | 0.001 |
| C8orf49 | SELP | hsa-mir-143 | 0.115612 | 0.001 |
| CALD1 | MAGI2-AS3 | hsa-mir-210 | 0.092006 | 0.001 |
| CALML3 | CMAHP | hsa-mir-141 | 0.063416 | 0.001 |
| CAMK2A | DLX6-AS1 | hsa-mir-145 | 0.04355 | 0.001 |
| CAMK2A | MEG3 | hsa-mir-205 | 0.05063 | 0.001 |
| CAMK2A | LINC00470 | hsa-mir-143 | 0.046935 | 0.001 |
| CLDN19 | C8orf49 | hsa-mir-429 | 0.054914 | 0.001 |
| CLDN19 | LINC00355 | hsa-mir-122 | 0.066559 | 0.001 |
| CLDN19 | FRMD6-AS2 | hsa-mir-182 | 0.076671 | 0.001 |
| CLMP | LINC00237 | hsa-mir-143 | 0.04611 | 0.001 |
| CLMP | LINC00314 | hsa-mir-143 | 0.046051 | 0.001 |
| CLMP | MEG3 | hsa-mir-508 | 0.033597 | 0.001 |
| CLRN1-AS1 | PDYN | hsa-mir-204 | 0.092119 | 0.001 |
| CLRN1-AS1 | C1orf141 | hsa-mir-211 | 0.057784 | 0.001 |
| CLRN1-AS1 | PPP1R12B | hsa-mir-489 | 0.031281 | 0.001 |
| CNGA2 | ATXN8OS | hsa-mir-210 | 0.062148 | 0.001 |
| CNGA2 | C8orf49 | hsa-mir-106a | 0.0544 | 0.001 |
| CNN1 | LINC00314 | hsa-mir-143 | 0.050269 | 0.001 |
| CPED1 | MEG3 | hsa-mir-205 | 0.045799 | 0.001 |
| CPED1 | CMAHP | hsa-mir-424 | 0.045204 | 0.001 |
| CSDC2 | LINC00491 | hsa-mir-145 | 0.04714 | 0.001 |
| CST5 | C10orf126 | hsa-mir-140 | 0.051325 | 0.001 |
| CXorf36 | WT1-AS | hsa-mir-106a | 0.062748 | 0.001 |
| DES | LINC00314 | hsa-mir-143 | 0.055041 | 0.001 |
| DGKB | WT1-AS | hsa-mir-145 | 0.154148 | 0.001 |
| DGKB | DIO3OS | hsa-mir-143 | 0.069191 | 0.001 |
| DIO3OS | MMRN2 | hsa-mir-508 | 0.031144 | 0.001 |
| DIO3OS | GLT1D1 | hsa-mir-215 | 0.069609 | 0.001 |
| DIO3OS | MRGPRF | hsa-mir-143 | 0.185155 | 0.001 |
| DIO3OS | ZEB1 | hsa-mir-143 | 0.104985 | 0.001 |
| DIO3OS | GLI2 | hsa-mir-508 | 0.041729 | 0.001 |
| DNM3OS | KRTAP4-4 | hsa-mir-145 | 0.086415 | 0.001 |
| DPYSL5 | POU6F2-AS2 | hsa-mir-383 | 0.050561 | 0.001 |
| DSCR10 | UGT1A1 | hsa-mir-424 | 0.088105 | 0.001 |
| DSCR4-IT1 | VRTN | hsa-mir-508 | 0.05006 | 0.001 |
| E2F2 | MAGI2-AS3 | hsa-mir-143 | 0.114608 | 0.001 |
| EDNRA | LINC00237 | hsa-mir-143 | 0.044551 | 0.001 |
| EDNRA | RMST | hsa-mir-195 | 0.039246 | 0.001 |
| EDNRA | LINC00491 | hsa-mir-145 | 0.046882 | 0.001 |
| EHD2 | LINC00470 | hsa-mir-424 | 0.05907 | 0.001 |
| EMCN | LINC00314 | hsa-mir-143 | 0.05372 | 0.001 |
| EMILIN1 | MEG3 | hsa-mir-508 | 0.036823 | 0.001 |
| FAM41C | TNS1 | hsa-mir-145 | 0.191925 | 0.001 |
| FBXL22 | C8orf49 | hsa-mir-143 | 0.055184 | 0.001 |
| FBXO40 | LINC00473 | hsa-mir-424 | 0.050298 | 0.001 |
| FBXO40 | BX255923.1 | hsa-mir-204 | 0.075579 | 0.001 |
| FKBP7 | MAGI2-AS3 | hsa-mir-210 | 0.102284 | 0.001 |
| FOXL2 | LINC00470 | hsa-mir-424 | 0.07324 | 0.001 |
| FRMD6-AS2 | CLDN19 | hsa-mir-182 | 0.111944 | 0.001 |
| FRMD6-AS2 | LHX3 | hsa-mir-182 | 0.042141 | 0.001 |
| GAGE1 | LINC00523 | hsa-mir-200a | 0.066922 | 0.001 |
| GAGE1 | NOVA1-AS1 | hsa-mir-489 | 0.070208 | 0.001 |
| GLI2 | LINC00113 | hsa-mir-145 | 0.064002 | 0.001 |
| GLI2 | C6orf99 | hsa-mir-140 | 0.036458 | 0.001 |
| GLI2 | DIO3OS | hsa-mir-508 | 0.041572 | 0.001 |
| GLT1D1 | DIO3OS | hsa-mir-215 | 0.056272 | 0.001 |
| GPR22 | MEG3 | hsa-mir-205 | 0.045296 | 0.001 |
| GUCY1A2 | CMAHP | hsa-mir-424 | 0.052736 | 0.001 |
| HAND2 | MEG3 | hsa-mir-508 | 0.029237 | 0.001 |
| HMCN2 | C20orf166-AS1 | hsa-mir-489 | 0.045233 | 0.001 |
| HOXD12 | ADARB2-AS1 | hsa-mir-205 | 0.068284 | 0.001 |
| HSPB7 | LINC00314 | hsa-mir-143 | 0.045802 | 0.001 |
| IRS4 | LINC00211 | hsa-mir-204 | 0.045064 | 0.001 |
| JAZF1-AS1 | EN1 | hsa-mir-205 | 0.039841 | 0.001 |
| JPH2 | LINC00314 | hsa-mir-143 | 0.058235 | 0.001 |
| JPH4 | LINC00314 | hsa-mir-143 | 0.056495 | 0.001 |
| JPH4 | LINC00470 | hsa-mir-143 | 0.044082 | 0.001 |
| KCNS2 | LINC00470 | hsa-mir-424 | 0.051067 | 0.001 |
| KIAA0087 | TMPRSS11A | hsa-mir-96 | 0.027287 | 0.001 |
| KIAA0087 | HSD17B13 | hsa-mir-195 | 0.052618 | 0.001 |
| KIAA1755 | LINC00470 | hsa-mir-424 | 0.043035 | 0.001 |
| KRT74 | AGAP11 | hsa-mir-205 | 0.044988 | 0.001 |
| KRT74 | DSCR4-IT1 | hsa-mir-211 | 0.073821 | 0.001 |
| KRT74 | LINC00458 | hsa-mir-204 | 0.048508 | 0.001 |
| KRTAP1-3 | LINC00314 | hsa-mir-204 | 0.054625 | 0.001 |
| KRTAP2-2 | LINC00314 | hsa-mir-204 | 0.068109 | 0.001 |
| KRTAP3-2 | LINC00314 | hsa-mir-204 | 0.055645 | 0.001 |
| KRTAP4-4 | DNM3OS | hsa-mir-145 | 0.142379 | 0.001 |
| KRTAP4-9 | LINC00314 | hsa-mir-204 | 0.051039 | 0.001 |
| KRTAP4-9 | LINC00051 | hsa-mir-205 | 0.046652 | 0.001 |
| LINC00052 | RPE65 | hsa-mir-145 | 0.086007 | 0.001 |
| LINC00052 | MAGEC2 | hsa-mir-363 | 0.046893 | 0.001 |
| LINC00052 | RTKN2 | hsa-mir-216b | 0.035097 | 0.001 |
| LINC00211 | SLITRK3 | hsa-mir-301b | 0.03609 | 0.001 |
| LINC00314 | BRS3 | hsa-mir-204 | 0.06444 | 0.001 |
| LINC00314 | FBXO40 | hsa-mir-211 | 0.042001 | 0.001 |
| LINC00314 | KRTAP4-9 | hsa-mir-204 | 0.055587 | 0.001 |
| LINC00314 | KRTAP3-2 | hsa-mir-204 | 0.069706 | 0.001 |
| LINC00314 | KRTAP11-1 | hsa-mir-204 | 0.04619 | 0.001 |
| LINC00314 | PAX1 | hsa-mir-106a | 0.062353 | 0.001 |
| LINC00314 | KRTAP2-2 | hsa-mir-204 | 0.071968 | 0.001 |
| LINC00314 | KRTAP1-3 | hsa-mir-204 | 0.068295 | 0.001 |
| LINC00314 | MYF5 | hsa-mir-106a | 0.053257 | 0.001 |
| LINC00355 | CLDN19 | hsa-mir-122 | 0.070711 | 0.001 |
| LINC00355 | LPP | hsa-mir-195 | 0.042034 | 0.001 |
| LINC00458 | TAS2R30 | hsa-mir-211 | 0.057263 | 0.001 |
| LINC00458 | UGT1A10 | hsa-mir-205 | 0.04347 | 0.001 |
| LINC00458 | UGT1A8 | hsa-mir-205 | 0.056269 | 0.001 |
| LINC00458 | LHX3 | hsa-mir-200a | 0.037188 | 0.001 |
| LINC00470 | CAMK2A | hsa-mir-143 | 0.106097 | 0.001 |
| LINC00483 | BPIFA1 | hsa-mir-216b | 0.057469 | 0.001 |
| LINC00523 | TCEAL6 | hsa-mir-141 | 0.068249 | 0.001 |
| LINC00523 | BRS3 | hsa-mir-508 | 0.063226 | 0.001 |
| LINC00523 | SPHKAP | hsa-mir-200a | 0.049543 | 0.001 |
| LINC00523 | GAGE1 | hsa-mir-200a | 0.076525 | 0.001 |
| LMOD1 | DLX6-AS1 | hsa-mir-145 | 0.07815 | 0.001 |
| LRRN4CL | LINC00470 | hsa-mir-424 | 0.059796 | 0.001 |
| MAGEC2 | C15orf54 | hsa-mir-182 | 0.031245 | 0.001 |
| MAGEC2 | KCNQ1DN | hsa-mir-195 | 0.060166 | 0.001 |
| MAGI2 | C2orf48 | hsa-mir-424 | 0.038502 | 0.001 |
| MAGI2-AS3 | E2F2 | hsa-mir-143 | 0.062779 | 0.001 |
| MAGI2-AS3 | MRGPRF | hsa-mir-106a | 0.061538 | 0.001 |
| MAGI2-AS3 | CALD1 | hsa-mir-210 | 0.073727 | 0.001 |
| MAGI2-AS3 | ABCC9 | hsa-mir-106a | 0.044168 | 0.001 |
| MAGI2-AS3 | FKBP7 | hsa-mir-210 | 0.089863 | 0.001 |
| MAGI2-AS3 | MRVI1 | hsa-mir-106a | 0.05805 | 0.001 |
| MAGI2-AS3 | PRKG1 | hsa-mir-210 | 0.048102 | 0.001 |
| MAMDC2 | LINC00314 | hsa-mir-143 | 0.046667 | 0.001 |
| MEF2C | WT1-AS | hsa-mir-145 | 0.171627 | 0.001 |
| MEG3 | NUDT10 | hsa-mir-508 | 0.056157 | 0.001 |
| MEG3 | AKT3 | hsa-mir-508 | 0.036517 | 0.001 |
| MEG3 | ZEB1 | hsa-mir-508 | 0.054771 | 0.001 |
| MEG3 | TMEM200B | hsa-mir-205 | 0.040681 | 0.001 |
| MEG3 | VWC2 | hsa-mir-508 | 0.052807 | 0.001 |
| MEG3 | ZEB1 | hsa-mir-205 | 0.062294 | 0.001 |
| MEG3 | CPED1 | hsa-mir-205 | 0.047004 | 0.001 |
| MEG3 | CAMK2A | hsa-mir-205 | 0.046561 | 0.001 |
| MEG3 | ZCCHC24 | hsa-mir-508 | 0.047691 | 0.001 |
| MEG3 | CPED1 | hsa-mir-508 | 0.046913 | 0.001 |
| MEG3 | NDN | hsa-mir-301b | 0.11207 | 0.001 |
| MEG3 | MRVI1 | hsa-mir-301b | 0.086603 | 0.001 |
| MEG3 | HAND2 | hsa-mir-508 | 0.037025 | 0.001 |
| MIR7-3HG | ZNF835 | hsa-mir-204 | 0.067378 | 0.001 |
| MRGPRF | MAGI2-AS3 | hsa-mir-106a | 0.061111 | 0.001 |
| MRGPRF | DIO3OS | hsa-mir-143 | 0.080023 | 0.001 |
| MRGPRF | LINC00491 | hsa-mir-145 | 0.035588 | 0.001 |
| MRVI1 | LINC00491 | hsa-mir-145 | 0.057741 | 0.001 |
| MRVI1 | MEG3 | hsa-mir-301b | 0.078471 | 0.001 |
| MRVI1 | MAGI2-AS3 | hsa-mir-106a | 0.060481 | 0.001 |
| MSRB3 | C2orf48 | hsa-mir-424 | 0.037056 | 0.001 |
| MUC2 | MYLK | hsa-mir-140 | 0.065502 | 0.001 |
| MYCT1 | WT1-AS | hsa-mir-106a | 0.062103 | 0.001 |
| MYLK | LINC00314 | hsa-mir-143 | 0.053569 | 0.001 |
| MYLK | LINC00470 | hsa-mir-143 | 0.047116 | 0.001 |
| MYLK | MUC2 | hsa-mir-140 | 0.052315 | 0.001 |
| MYOCD | FAM41C | hsa-mir-145 | 0.079526 | 0.001 |
| MYOG | LINC00211 | hsa-mir-301b | 0.040576 | 0.001 |
| NAV2-AS2 | BPIFA1 | hsa-mir-204 | 0.065896 | 0.001 |
| NAV2-AS2 | PRKG1 | hsa-mir-182 | 0.047267 | 0.001 |
| NDN | MEG3 | hsa-mir-301b | 0.089095 | 0.001 |
| NDN | LINC00470 | hsa-mir-424 | 0.048859 | 0.001 |
| NEUROD1 | AGAP11 | hsa-mir-204 | 0.048219 | 0.001 |
| NEXN | LINC00314 | hsa-mir-143 | 0.056783 | 0.001 |
| NKX2-1-AS1 | PHOX2B | hsa-mir-301b | 0.051204 | 0.001 |
| NOVA1-AS1 | TRDN | hsa-mir-489 | 0.068781 | 0.001 |
| NOVA1-AS1 | SPHKAP | hsa-mir-489 | 0.0458 | 0.001 |
| NOVA1-AS1 | GAGE1 | hsa-mir-489 | 0.062704 | 0.001 |
| NPFFR2 | ATXN8OS | hsa-mir-204 | 0.065296 | 0.001 |
| NUDT10 | MEG3 | hsa-mir-508 | 0.047246 | 0.001 |
| OGN | LINC00470 | hsa-mir-424 | 0.044554 | 0.001 |
| OSR2 | C2orf48 | hsa-mir-424 | 0.046437 | 0.001 |
| PAX1 | LINC00314 | hsa-mir-106a | 0.056771 | 0.001 |
| PDLIM3 | LINC00314 | hsa-mir-143 | 0.061127 | 0.001 |
| PDYN | CLRN1-AS1 | hsa-mir-204 | 0.120434 | 0.001 |
| PLN | C8orf49 | hsa-mir-143 | 0.100452 | 0.001 |
| PLN | WT1-AS | hsa-mir-106a | 0.091697 | 0.001 |
| PLN | LINC00314 | hsa-mir-143 | 0.069011 | 0.001 |
| PLN | LINC00473 | hsa-mir-195 | 0.067178 | 0.001 |
| POU6F2-AS2 | DPYSL5 | hsa-mir-383 | 0.039226 | 0.001 |
| PPP1R12B | AGAP11 | hsa-mir-424 | 0.068012 | 0.001 |
| PPP1R12B | RMST | hsa-mir-195 | 0.041459 | 0.001 |
| PPP1R12B | CLRN1-AS1 | hsa-mir-489 | 0.036928 | 0.001 |
| PPP1R12B | WT1-AS | hsa-mir-363 | 0.055369 | 0.001 |
| PRR9 | LINC00458 | hsa-mir-204 | 0.049393 | 0.001 |
| PTGER3 | LINC00470 | hsa-mir-143 | 0.046948 | 0.001 |
| PTGER3 | C8orf49 | hsa-mir-143 | 0.050233 | 0.001 |
| PTPRB | WT1-AS | hsa-mir-106a | 0.058518 | 0.001 |
| RERG-IT1 | PBX1 | hsa-mir-182 | 0.045367 | 0.001 |
| RERG-IT1 | BRS3 | hsa-mir-182 | 0.053834 | 0.001 |
| RGS21 | CLRN1-AS1 | hsa-mir-211 | 0.0464 | 0.001 |
| RHOJ | LINC00470 | hsa-mir-424 | 0.042904 | 0.001 |
| RMST | DPYSL5 | hsa-mir-205 | 0.036692 | 0.001 |
| ROBO4 | LINC00237 | hsa-mir-143 | 0.042533 | 0.001 |
| RPE65 | LINC00052 | hsa-mir-145 | 0.059015 | 0.001 |
| RUNX1T1 | LINC00491 | hsa-mir-145 | 0.039155 | 0.001 |
| RUNX1T1 | LINC00470 | hsa-mir-424 | 0.056484 | 0.001 |
| SELP | C8orf49 | hsa-mir-143 | 0.063642 | 0.001 |
| SEZ6L | DSCR4-IT1 | hsa-mir-211 | 0.047708 | 0.001 |
| SFRP4 | LINC00470 | hsa-mir-424 | 0.051757 | 0.001 |
| SORCS3 | ADARB2-AS1 | hsa-mir-195 | 0.040317 | 0.001 |
| SOX21 | SOX21-AS1 | hsa-mir-301b | 0.045209 | 0.001 |
| SPARCL1 | MAGI2-AS3 | hsa-mir-106a | 0.038385 | 0.001 |
| SPARCL1 | AL713998.1 | hsa-mir-141 | 0.038917 | 0.001 |
| SPHKAP | NOVA1-AS1 | hsa-mir-489 | 0.049167 | 0.001 |
| SPHKAP | LINC00523 | hsa-mir-200a | 0.048167 | 0.001 |
| SPRR2G | ADARB2-AS1 | hsa-mir-205 | 0.062906 | 0.001 |
| STXBP5L | LINC00488 | hsa-mir-216b | 0.031408 | 0.001 |
| SYNPO2 | WT1-AS | hsa-mir-363 | 0.047682 | 0.001 |
| SYNPO2 | C2orf48 | hsa-mir-424 | 0.032424 | 0.001 |
| SYTL4 | MUC2 | hsa-mir-145 | 0.036619 | 0.001 |
| TAS2R30 | LINC00458 | hsa-mir-211 | 0.0683 | 0.001 |
| TCEAL6 | LINC00523 | hsa-mir-141 | 0.056359 | 0.001 |
| TCEAL7 | WT1-AS | hsa-mir-106a | 0.065237 | 0.001 |
| TCF23 | AGAP11 | hsa-mir-424 | 0.067779 | 0.001 |
| TCF23 | FAM41C | hsa-mir-145 | 0.072883 | 0.001 |
| TCF23 | LINC00470 | hsa-mir-143 | 0.043073 | 0.001 |
| TMEM200A | MEG3 | hsa-mir-508 | 0.02784 | 0.001 |
| TMEM200B | MEG3 | hsa-mir-205 | 0.049602 | 0.001 |
| TMEM200B | DIO3OS | hsa-mir-143 | 0.081445 | 0.001 |
| TMPRSS11A | C10orf126 | hsa-mir-141 | 0.049527 | 0.001 |
| TNS1 | FAM41C | hsa-mir-145 | 0.082492 | 0.001 |
| TNS1 | AC020907.1 | hsa-mir-424 | 0.049355 | 0.001 |
| TPTE | LINC00051 | hsa-mir-363 | 0.047716 | 0.001 |
| TRDN | NOVA1-AS1 | hsa-mir-489 | 0.059228 | 0.001 |
| TSHZ3 | LINC00470 | hsa-mir-424 | 0.06578 | 0.001 |
| UGT1A1 | DSCR10 | hsa-mir-424 | 0.086862 | 0.001 |
| UGT1A10 | LINC00458 | hsa-mir-205 | 0.043013 | 0.001 |
| UGT1A8 | LINC00458 | hsa-mir-205 | 0.065927 | 0.001 |
| VIPR2 | LINC00470 | hsa-mir-424 | 0.042905 | 0.001 |
| VRTN | DSCR4-IT1 | hsa-mir-508 | 0.042518 | 0.001 |
| VRTN | C8orf49 | hsa-mir-106a | 0.057327 | 0.001 |
| VWC2 | MEG3 | hsa-mir-508 | 0.043955 | 0.001 |
| VWC2 | KIAA0087 | hsa-mir-141 | 0.035675 | 0.001 |
| WFDC5 | C8orf49 | hsa-mir-301b | 0.054508 | 0.001 |
| WT1-AS | ASPN | hsa-mir-363 | 0.03927 | 0.001 |
| WT1-AS | CXorf36 | hsa-mir-106a | 0.042236 | 0.001 |
| WT1-AS | CACNB2 | hsa-mir-145 | 0.068521 | 0.001 |
| WT1-AS | PPP1R12B | hsa-mir-106a | 0.040379 | 0.001 |
| WT1-AS | TMEM200B | hsa-mir-363 | 0.045723 | 0.001 |
| WT1-AS | LMOD1 | hsa-mir-363 | 0.03273 | 0.001 |
| WT1-AS | CLEC14A | hsa-mir-145 | 0.143889 | 0.001 |
| WT1-AS | TNS1 | hsa-mir-106a | 0.049424 | 0.001 |
| WT1-AS | DGKB | hsa-mir-145 | 0.146178 | 0.001 |
| WT1-AS | PLN | hsa-mir-363 | 0.05174 | 0.001 |
| WT1-AS | PLN | hsa-mir-106a | 0.074556 | 0.001 |
| WT1-AS | TMEM200A | hsa-mir-106a | 0.054504 | 0.001 |
| WT1-AS | PPP1R12B | hsa-mir-363 | 0.040808 | 0.001 |
| ZCCHC24 | MEG3 | hsa-mir-508 | 0.046042 | 0.001 |
| ZCCHC24 | LINC00237 | hsa-mir-143 | 0.055599 | 0.001 |
| ZCCHC24 | LINC00470 | hsa-mir-424 | 0.051105 | 0.001 |
| ZEB1 | MEG3 | hsa-mir-508 | 0.055616 | 0.001 |
| ZEB1 | MEG3 | hsa-mir-205 | 0.057217 | 0.001 |
| ZEB1 | DIO3OS | hsa-mir-143 | 0.057385 | 0.001 |
| ZNF835 | MIR7-3HG | hsa-mir-204 | 0.041076 | 0.001 |
