# Supplementary figures and images for: Integrated Bioinformatic Analysis of a Competing Endogenous RNA Network Reveals a Prognostic Signature in Endometrial Cancer
Source: Front Oncol. 2019 May 29;9:448. doi: 10.3389/fonc.2019.00448 (PMC6549402; doi:10.3389/fonc.2019.00448)

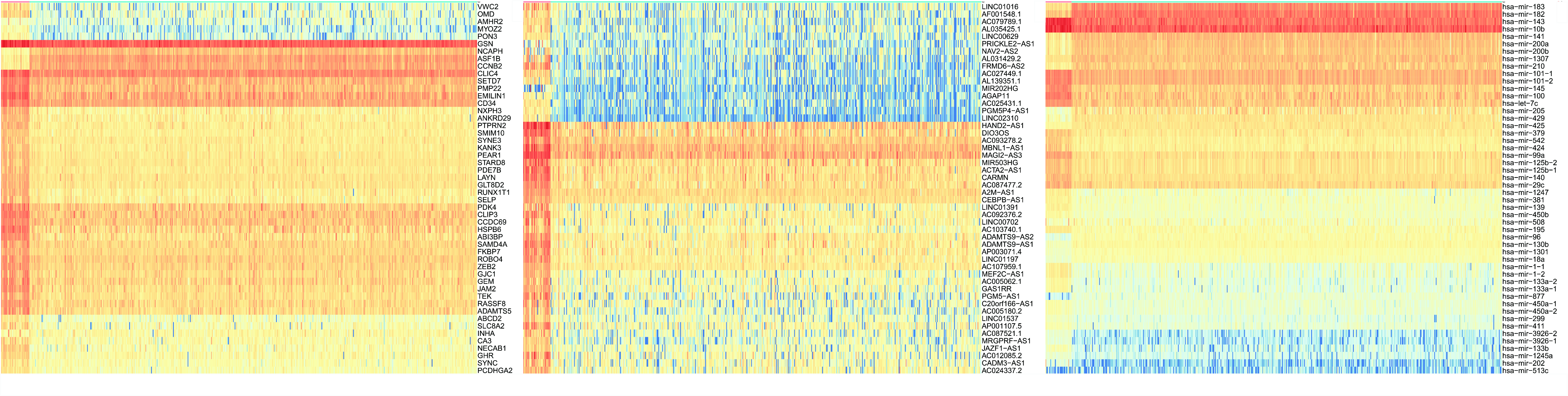

Supplement: Supplement Figure 2 — All lncRNA node degree analysis reveals specific properties of the ceRNA network. [file Image_1.TIF]
